# PD-L1 immunohistochemistry assay optimization to provide more comprehensive pathological information in classic Hodgkin lymphoma

**DOI:** 10.1007/s12308-023-00530-1

**Published:** 2023-02-01

**Authors:** Yunfei Shi, Lan Mi, Yumei Lai, Min Zhao, Ling Jia, Tingting Du, Yuqin Song, Xianghong Li

**Affiliations:** 1 Key Laboratory of Carcinogenesis and Translational Research (Ministry of Education/Beijing),department of Pathology, Peking University Cancer Hospital & Institute, Beijing, China; 2 Key Laboratory of Carcinogenesis and Translational Research (Ministry of Education/Beijing),department of Lymphoma, Peking University Cancer Hospital & Institute, Beijing, China

**Keywords:** Hodgkin Lymphoma, PD-L1, Immunohistochemistry, RNAscope, Immunotherapy

## Abstract

Overexpression of PD-L1 can be a predictive marker for anti-PD-1 therapeutic efficacy in classic Hodgkin lymphoma (CHL); however, harmonization of different IHC assays remains to be accomplished, and interpretations of PD-L1 immunostaining results remain controversial in CHL. In this study, we sought to optimize the PD-L1 immunohistochemistry (IHC) assay in CHL. All tests were performed on a tumour tissue microarray established from 54 CHL cases. Three IHC antibodies (405.9A11, SP142, 22C3) for detecting PD-L1 expression were compared semi quantitatively with the RNAscope assay (No. 310035, ACD), and the difference in the expression in background immune cells (ICs) between assays and the associations of expression levels with densities of TILs/TAMs were also analysed. 405.9A11 demonstrated best specificity in HRS cells and best sensitivity in ICs. Positive expression of PD-L1 was more frequent in ICs (85.2%) than in HRS cells (48.1%). Different subgroups of background ICs, including tumour-associated macrophages (TAMs), were assessed and scored for CD4, CD8, FOXP3, and CD163 expression. PD-L1 expression on ICs was the factor most associated with the density of TAMs. 405.9A11 provided the most convincing PD-L1 expression results. Pathologists should report PD-L1 expression in a combined manner, including both the status of HRS cells and the percentage of PD-L1-positive ICs.

**Supplementary Information:**

The online version contains supplementary material available at 10.1007/s12308-023-00530-1.

## Introduction

Classical Hodgkin lymphoma (CHL) is characterized by a minority of malignant Hodgkin and Reed–Sternberg cells (HRS cells) within an overwhelming background of ineffective inflammatory infiltrates [1]. Although most CHL cases are curable, treating relapsed or refractory (R/R) CHL cases is still challenging.

The programmed death-1 (PD-1) blocking antibody nivolumab and its mimics [2], the so-called immune checkpoint inhibitors, can promote and stimulate an antitumour effect via the host immune system rather than directly targeting malignant cells; such agents have been shown have substantial therapeutic activity in R/R CHL [3–5]. PD-1, one of the most important immune checkpoints [6], is reported to be expressed in peritumoral activated T cells [7] rather than HRS cells [8] in CHL. PD-1 has two ligands: programmed death-ligand 1 (PD-L1) and PD-L2 [9]. Binding of PD-L1/PD-L2 to PD-1 delivers an inhibitory signal that inhibits the overt physical activation of T cells and prevents tumour escape from host immune control [8, 10, 11]. Both PD-L1 and PD-L2 can have genetic alterations in CHL [12]. The expression of PD-L1 and PD-L2 can be measured by immunohistochemistry (IHC) staining of formalin-fixed paraffin embedded (FFPE) tissue sections [13–16]. The incidence of aberrant PD-L2 expression is similar to [12] or less common than [16, 17] that of aberrant PD-L1 expression, and there are currently more commercial antibodies (Abs) and assays for targeting PD-L1 available [18]. As such, we focused on tests for PD-L1, which have been proposed as complementary for determining the probability of benefit from anti-PD-1/L1 agents [19].

The expression of PD-L1 can be found not only on tumour cells but also on peritumoral immune cells (ICs) in various tumours, including lymphoma [20]. The expression level of PD-L1 on tumour cells and ICs can serve as a predictor of therapeutic efficacy [20, 21]. In CHL, overexpression of PD-L1 can be a predictive marker for anti-PD-1 therapeutic efficacy [8, 15]; however, harmonization of different IHC assays remains to be accomplished, and interpretations of PD-L1 immunostaining results remain controversial [16, 21, 22]. Recently, the RNAscope assay, which employs in situ hybridization of FFPE tumour samples [23–25], might be a promising method for assessing PD-L1 mRNA levels in various types of solid tumours [25–27] to provide for the PD-L1 expression level independent of IHC assays in melanoma [27]; however, this method has rarely been described in CHL.

In various solid tumours, PD-L1 expression has been found to be related to the density of certain subtypes of ICs, including CD4+ T helper cells (Ths), CD8+ cytotoxic T cells (CTLs), FOXP3+ regulatory T cells (Tregs), and CD163+ tumour-associated macrophages (TAMs) [28–32]. TAMs are correlated with poor prognosis in CHL [33, 34]. TAMs can express PD-L1 under the activation of IFN-γ- and PD-1-positive T-cell infiltration [35] or may gain PD-L1 expression via trogocytosis of HRS cells [36]. Thus, the association between PD-L1 expression and the densities of different types of ICs need further investigation in CHL.

In this study, we sought to (1) determine the best Ab and assay for detecting PD-L1 by independently comparing three IHC assays with the RNAscope assay; (2) provide a more accurate expression status of PD-L1 in CHL, on both HRS cells and ICs; and (3) analyse the association between PD-L1 expression and different elements of background ICs.

## Materials and methods

### Patient selection and tissue microarrays

The FFPE specimens of 54 nodal tissues from patients diagnosed with CHL were retrieved from the Department of Pathology at our Hospital. All cases were reviewed by two hematopathology experts to confirm the pathological diagnosis. The tissue microarrays (TMAs) for subsequent experiments were created by retrieving duplicate cores (1 mm in diameter) from representative areas in each reviewed block using an arrayer (Alphelys, Plaisir, France) as described previously [37].

### Immunol staining and evaluation of the expression of PDL1 and other associated biomarkers

The expression of PD-L1 was assessed with 3 different Abs and assays: clone 405.9A11 (referred to as 9A11, Cell Signalling Technology), SP142 (Ventana, Roche) [38], and 22C3 (Dako, Agilent Technologies) [39]. Antibodies against PD-1 (UMAB199, Origen), CD4 (EP204, Origen), FOXP-3 (ab20034, Abcam), CD8 (SP16, BioCare), and CD163 (NCL-CD163, Novocastra) were also used. The dilutions were 1:50 or those indicated in the instructions from each supplier. All IHC staining steps were performed on an automated IHC staining instrument (VENTANA, Roche) excluding the staining for clone 22C3 (PD-L1), which was performed with a Dako Auto Stainer, and optimization for each antibody was performed for minimum nonspecific staining by adjusting the primary antibody concentration and reagent incubation times. Negative/positive controls were established as recommended [39]. The immunohistochemistry assays for the 3 different clones of anti-PDL1 Abs were performed according to previously published methods [38, 40, 41]; a description of the IHC assay methodology is further described in the supplementary materials.

#### Pathological evaluation

After staining, the expression levels of PD-L1 and the other abovementioned markers were independently reviewed in six representative fields at high power (×400) magnification from 2 different TMA cores. PD-L1 staining was considered positive if moderate/strong staining (yellow to brown signal located) of the membrane and/or cytoplasm was seen on target cells (either HRS cells or ICs), and the threshold for positive PD-L1 expression in HRS cells was >25% [16]. PD-L1 expression in ICs was calculated as the proportion of the tumour area occupied by PD-L1-positive ICs. IC staining in the tumour microenvironment, including the pattern of staining (aggregates or single cells dispersed among TCs) and the type of stained immune cell (lymphocytes, macrophages, dendritic cells, and granulocytes), was evaluated [38, 42]. The tumour area was defined as the area containing viable TCs, their associated intratumoral stroma and contiguous peritumoral stroma; the threshold for a positive value for ICs was >10% [43, 44].

##### Grading

Scoring of PD1+, CD4+, FOXP3+, CD8+, and CD163+ cells was performed independently by two pathologists based on visual estimation in reference to methods in previous studies [33, 45, 46]. The relative percentage of cells that stained positive on IHC in tumour cell regions in overall cells was calculated as an average of the values the duplicate cores and graded as follows: <5% (score 1+), 5–25% (score 2+), and >25% (score 3+). PD-1 was considered positive if the percentage of PD-1+ cells to total cells was ≥10% [47, 48].

### In situ hybridization to detect EBV-encoded RNA

EBV status was determined by in situ hybridization to detect EBV-encoded RNA 1 and 2 (EBER1/2s) using peroxidase-labelled probes (ISH-7001UM, Beijing Zhongshan Golden Bridge Biotechnology). Tissue from a known EBV-positive CHL case was used as a positive control. The results were independently dual-assessed. The EBV status was considered positive if at least one definitive HRS cell expressed EBER [49].

### In situ hybridization to detect PD-L1 mRNA and scoring guidelines

In situ detection of PD-L1 transcripts in the CHL TMA samples was performed using the RNAscope Detection Kit (Cat. No. 310035, ACD, USA) with custom-designed horseradish peroxidase (HRP)-labelled probes (from ACD, USA). Briefly, 5 μm TMA sections were deparaffinized, boiled with preamplification reagent for 15 min, and subjected to protease digestion followed by hybridization for 2 hours with target probes against PD-L1 mRNA. Detection reagents (DAB substrate and solutions) were subsequently pipetted onto the tissue sections to detect hybridization signals, enabling RNA molecules to be visualized as brown chromogenic dots, and the slides were ultimately counterstained with haematoxylin. DapB and PPIB probes were used as negative and positive control probes, respectively.

#### Grading

A manual semi-quantitative scoring system for PD-L1 mRNA was established according to the estimated number of punctate dots present within the boundary of each HRS cell at 40× magnification: the scores were defined as 0 (less than 1 dot/cell), 1+ (1–4 dots/cell), 2+ (5–10 dots/cell without dot clusters), or 3+ (>10 dots/cell or with dot clusters) [27]. All samples were interpreted in a double-blind manner. Positive PD-L1 mRNA expression on the background ICs could have served as an internal positive control, but it was difficult to estimate the percentage of area occupied by positive ICs for the “dot-like” staining pattern.

### Statistical analysis

Descriptive statistics were used to summarize covariates. Categorical covariates are reported as percentages and counts. Continuous variables are reported as medians and ranges. Pearson’s chi-square test was used to analyse categorical covariates. Student’s *t* (normal distribution) and Mann–Whitney *U* (non-normal distribution) tests were used to analyse continuous covariates.

The comparisons of PD-L1 protein expression between each of the 3 different IHC assays and RNAscope and the associations of PD-L1 expression with other characteristics (including PD-1 expression, EBERs levels, and TIL/TAM marker density scores) were analysed using the chi-square test (or Fisher’s exact test when necessary), and the correlations were determined using Pearson’s correlation coefficient (*r*). The Kappa index was determined in all patients [K-Index = (K-FLC × sALB) / (sFLC-K × CSF-ALB)] to evaluate the precision of each diagnostic pathology method. Concordance values between mRNA levels and antibodies were assessed by Cohen’s kappa, which was calculated as an index of interrater agreement. We used the following scale: < 0.50: low concordance; 0.50–0.75: moderate concordance; 0.75–0.90: high concordance; and > 0.9: nearly perfect concordance [27]. All statistical tests were two-sided with an alpha level of 0.05 as the significance cut-off value. All analyses were performed in statistical software R 4.1.3 (NYC, co.). Survival analysis on this relatively small series of CHL cases was also done and discussed in the suppletory materials.

## Results

### Clinical and pathological features

All 54 CHL patients primarily presented with nodal involvement. The mean onset age was 44.3 years (range: 22.0~68.0 years, median age: 45.5 years), and the male:female ratio was 1.7:1 (34/20). Of the 45 patients with hospitalization data available, 40.0% (18/45) had B symptoms, and 53.3% (24/45) were staged as stage III~IV. All 45 patients were administered ABVD (doxorubicin, bleomycin, vinblastine, and dacarbazine) as their first-line treatment, combined with other therapies when necessary: 7 patients received treatment combined with radiotherapy, 3 patients underwent sequential autologous stem cell transplantation (ASCT), and 1 patient who relapsed was treated with chimeric antigen receptor T-cell (CAR-T) immunotherapy. All CHL cases were classified according to the WHO 2017 classification of lymphoma: 59.3% (32/54) of cases were classified as nodular sclerosis (NS) type, 38.9% (21/54) of cases were classified as mixed cellularity (MC) type and 1 (1.9%) case was classified as lymphocyte-rich (LR) type. The total positive rate of EBER1/2s was 25.9% (14/54): 9.4% (3/32) of the NS type cases, 51.4% (11/21) of the MC type cases, and 0% (0/1) of the LR type cases. There was a significant difference (*P*=0.002) in EBV status among the different types.

### Evaluation of PD-L1 expression in HRS cells and ICs by immunostaining with different antibody clones

**Fig. 1:**
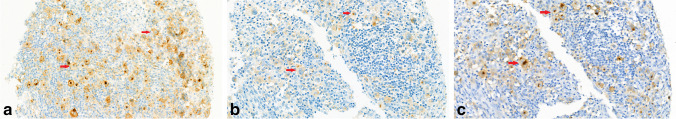
Positive expression of PD-L1 on the cell membrane of HRS cells detected by immunostaining with different clones of antibodies. There were difference in decorating the positive cell membrane and more background IC cells for positive for PD-L1 in stained by 9A11 (**1A**), SP142 (**1B**), and 22C3 (**1C**) from high power field of the same CHL case and tissue core under microscope (red arrows). Figure 1D–1F shows prominent positive expression of PD-L1 on the background immune cells (ICs, black arrows) from the same CHL case of HPF with 9A11 (**1D**), SP142 (**1E**), and 22C3 (**1F**); in this case, the HRS cells were negative for PD-L1, and there were obviously more PD-L1-positive background IC cells stained with 9A11 than other assays from low power field of the whole tissue core (on the upper right). The blue arrow in Fig. 1D indicated no staining on internal negative control like microvascular endothelial cell to ensure no unspecific background staining.

**Fig. 2:**
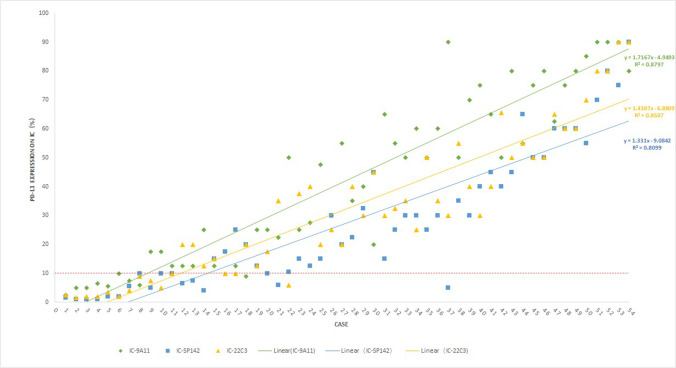
Comparison for of PD-L1 level expression (%) on background immune cells (ICs) in CHL, by IHC with different assays. All 54 cases were ranked and coordinated according to the mean value of the percentage of PD-L1-positive IC cells of each case; scoring IC by 9A11 shows the best sensitivity and linear correlation.

The IF assessment focused on HRS cells. The PD-L1-positive rate was 48.1% (26/54) for 9A11, 59.3% (32/54) for SP142, and 63.0% (34/54) for 22C3. All three antibodies could delineate the cell membrane of the positive cells, but there was still a difference (Fig. 1A, 1B, 1C) [50]. For the background ICs, there were inconsistencies: the percentage of cells with positive PD-L1 expression ranged from 2.5 to 90.0% (mean 42.3%) for 9A11, 1.0 to 90.0% (mean 27.5%) for SP142, and 1.5 to 90.0% (mean 32.5%) for 22C3 (Fig. 1D, 1E, 1F). Moreover, the rate of positive PD-L1 expression in ICs (>10% as the cut-off value) was 85.2% (46/54) for 9A11, 68.5% (37/54) for SP142, and 74.1% (40/54) for 22C3 (Fig. 2).

### PD-L1 mRNA level in HRS cells and its correlation with PD-L1 immunostaining

**Fig. 3:**
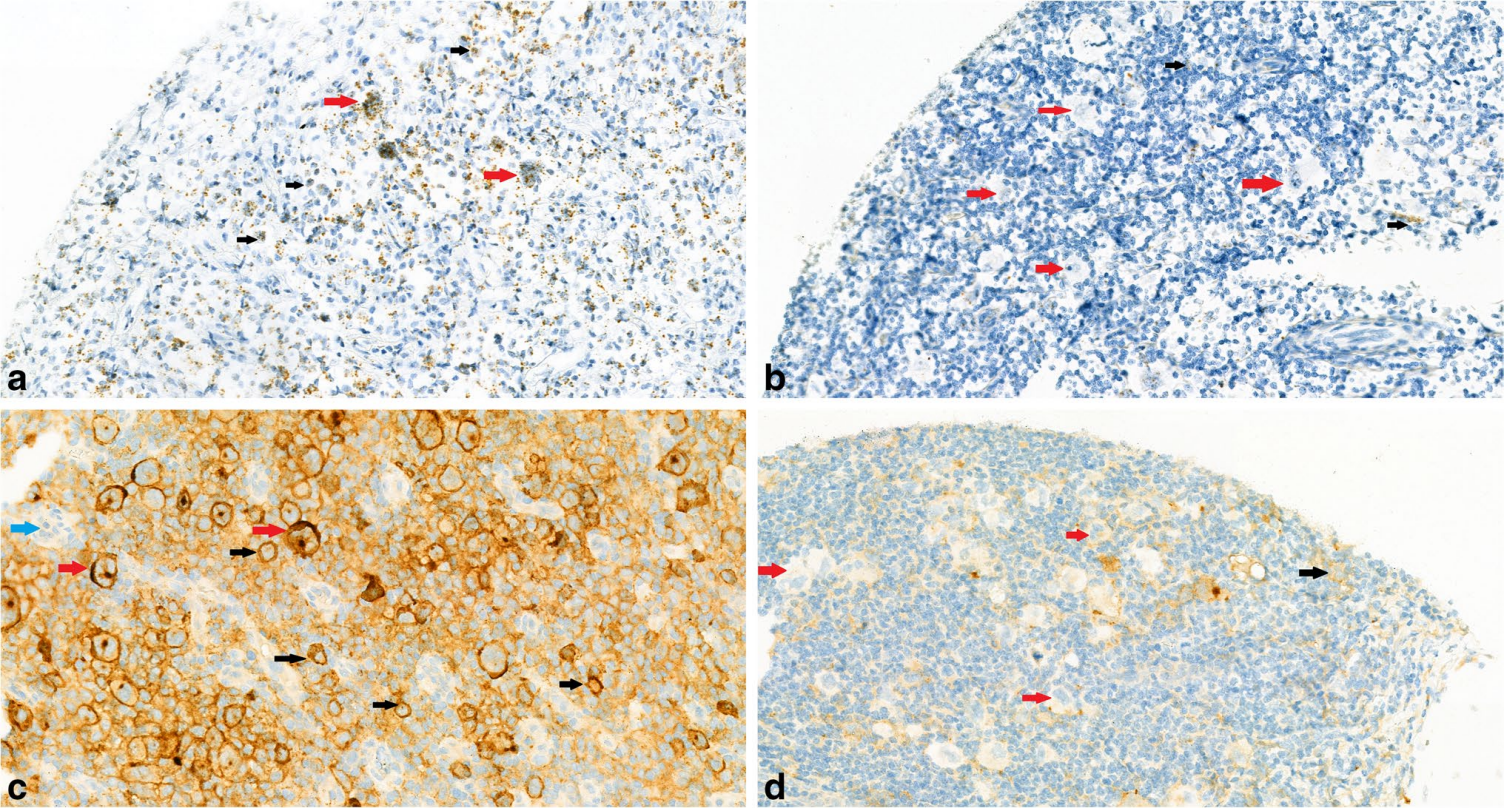
PD-L1 mRNA expression detected by RNAscope in HRS cells. **3A** Showed a case with high level expression on HRS cells (scored 3+, red arrows) and **3B** indicated another case with low level of expression on HRS cells (scored 1+, red arrows); please note some background cell show relatively high level of expression in **3B** (black arrows). The PDL1 immunostaining by 9A11assay showed the best concordance with mRNA expression on HRS cells: **3C** showed PD-L1 protein positive on HRS cell (red arrows) of the same case with **3A**, and **3D** was PD-L1 negative on HRS cells and was from the same case of **3B**. The black arrows indicated the PD-L1 + non-malignant cells in all figures, and the blue arrow in Fig. 3C indicated no staining on internal negative control like microvascular endothelial cell to ensure no unspecific background staining.

PD-L1 mRNA expression in HRS cells was detected successfully in 46 cases, and 45.7% (21/46) of cases expressed PD-L1 mRNA at higher levels (2+~3+, Fig. 3A, 16 cases had 3+ PD-L1 mRNA expression; 5 cases had 2+ PD-L1 mRNA expression). The other 54.3% (25/46) of cases expressed PD-L1 mRNA at a lower level (0~1+, Fig. 3B, 23 cases had 1+ PD-L1 mRNA expression; 2 cases had a PD-L1 mRNA expression score of 0). According to the IHC assays of the same 46 abovementioned cases with the 9A11, SP142, and 22C3 Abs, 21 cases, 26 cases, and 29 cases showed positivity on HRS cells. Of these positive cases, 95.23% (20/21), 76.92% (20/26), and 72.41% (21/29) expressed PD-L1 mRNA at higher levels (2+~3+); for the remaining PD-L1 protein-negative cases, 96.0% (24/25), 95.0% (19/20), and 100% (17/17) of cases expressed mRNA at lower levels (0~1+).

**Fig. 4:**
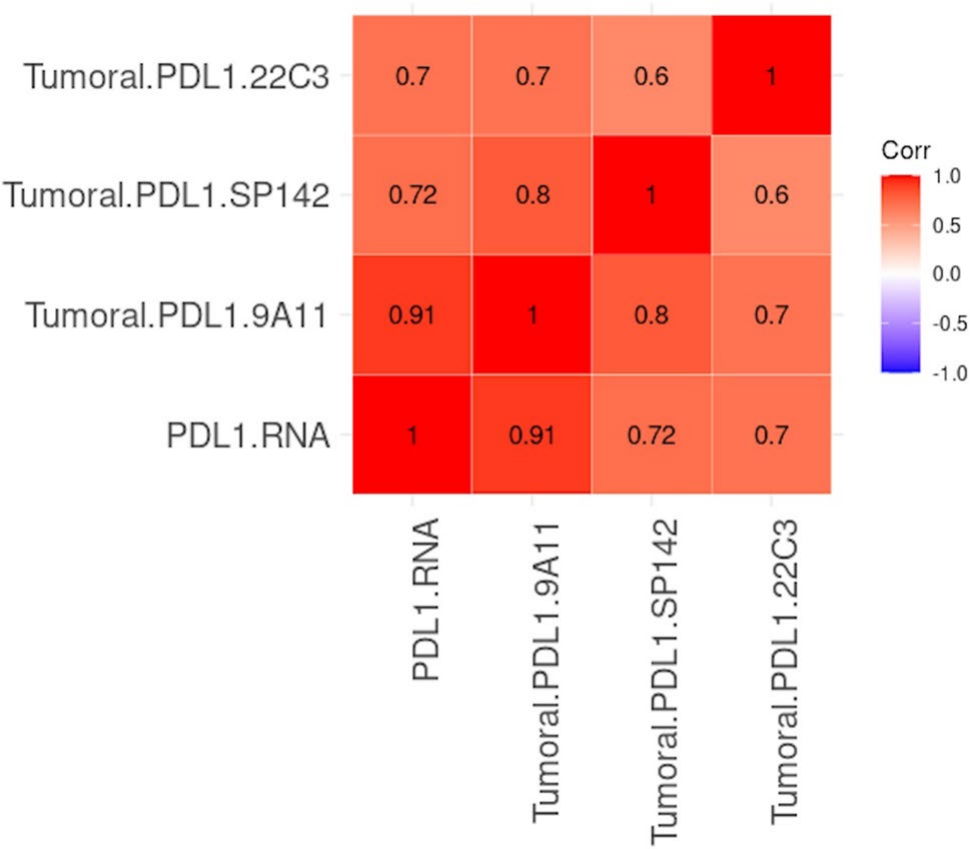
Comparison the concordance of PD-L1 expression on HRS cells (tumoral PD-L1) between protein level detected by with 3 different clones of Antibodies and mRNA level detected by RNAscope as independent reference platform. Assessed by Cohen’s Kappa that was calculated as an index of inter-rater agreement.

9A11 showed the best linear correlation with PD-L1 mRNA level in HRS cells, with a kappa index of 0.91 (nearly perfect concordance, see Fig. 3A vs. Fig. 3C and Fig. 3B vs. Fig. 3D); the kappa index was 0.72 for SP142 and 0.70 for 22C3 (both showing moderate concordance), as shown in Fig. 4.

### Scoring tumoral immune cell subpopulation-associated biomarkers, including PD-1, in CHL

When we scored the different groups of background immune cells by IHC (Fig. S1A–1D & Fig. S1F–S1I), we obtained the following results: CD4+ Ths: 3 cases were scored as 1+, 33 cases were scored as 2+, and 18 cases were scored as 3+; FOXP3+ Tregs: 9 cases were scored as 1+, 42 cases were scored as 2+, and 3 cases were scored as 3+; CD8+ CTLs: 1 case was scored as 1+, 34 cases were scored as 2+, and 19 cases were scored as 3+; CD163+ TAMs: 12 cases were scored as 1+, 34 cases were scored as 2+, and 8 cases were scored as 3+. See Table 1 for details. The percentage of PD-1+ cells ranged from 0.0 to 90.0% (mean 7.41%, Fig. S1E and S1J), and 25.9% (14/54) of cases were considered “positive” (cut-off value ≥10%).

**Table 1:** Details for scoring of the background immune cells (ICs) with associated biomarkers in CHL (*n*=52)

	Density and scoring of ICs cells				
	Percentage range (%)	Average (%)	1+(%)	2+(%)	3+(%)
CD4+Ths	3.0~60.0	21.4	3 (5.6)	33 (61.1)	18(33.3)
FOXP3+Tregs	0.5~40.0	12.6	9 (16.7)	42 (77.8)	3(5.6)
CD8+ CTLs	3.0~65.0	22.0	1 (1.9)	34 (63.0)	19(35.2)
CD163+TAMs	1.0~50.0	15.2	12 (22.2)	34 (63.0)	8(14.8)

CD4+ Ths (T helper cells), CD8+ CTLs (cytotoxic T cells), FOXP3+ regulatory T cells (Tregs), and CD163+ TAMs (tumor-associated macrophages). The relative percentage of positively stained ICs by certain makers in relation to overall cellularity in tumor cell regions was calculated and grade IHC score as: <5%= 1+, 5–25%= score 2+, >25% = score 3+.

### Correlation between PD-L1 expression and densities of different subpopulations of ICs

**Fig. 5:**
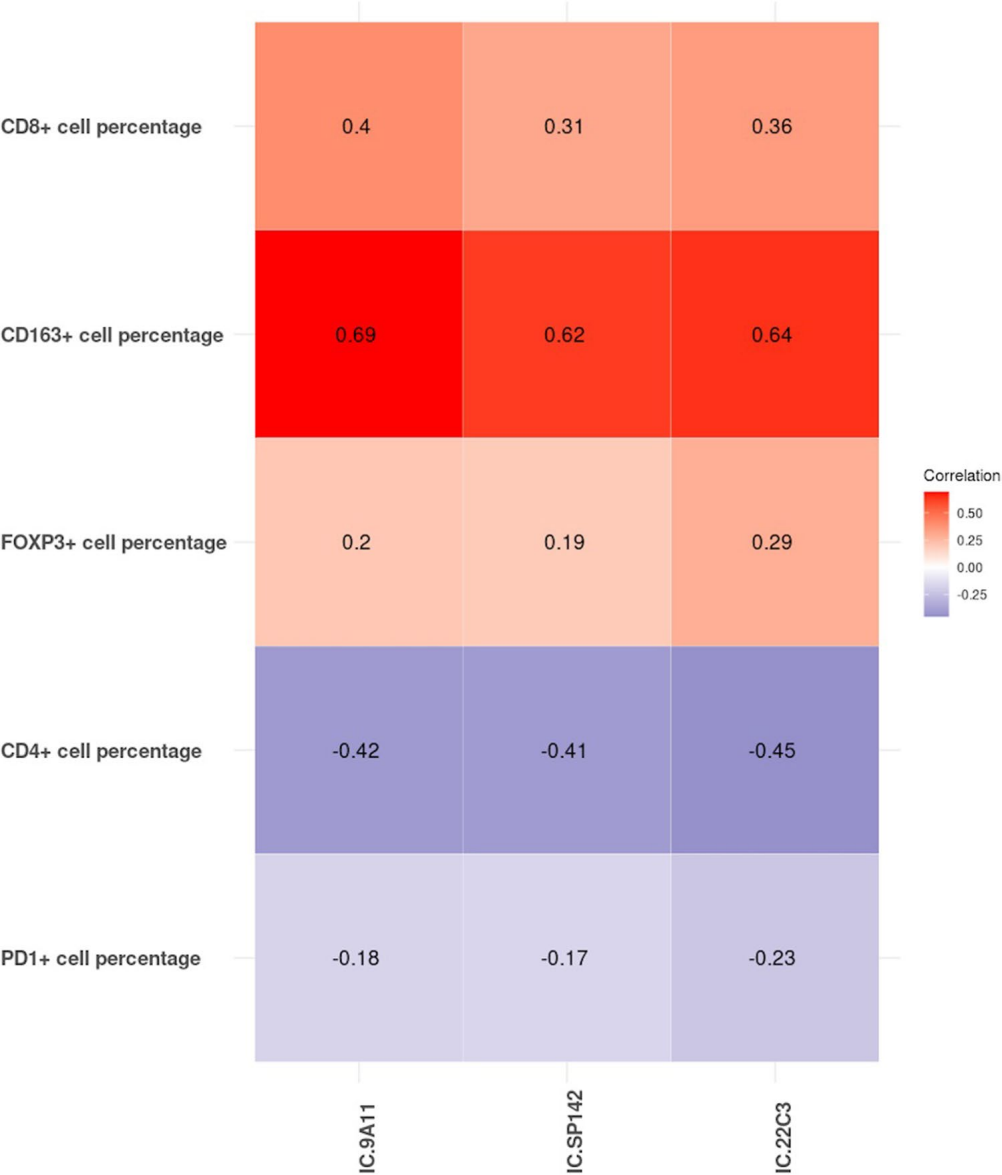
Comparison between the concordance of PD-L1 expression percentage on background immune cells (ICs) with different assays and the densities of different subgroups of immune cells. Relationships were determined using Pearson’s correlation coefficient (*r*).

If considering PD-L1 expression on HRS cells, PD-L1 positivity showed poor concordance with the scores of IC subpopulations for CD163, CD4, CD8, and FOXP3 staining (Fig. S2). The PD-1 positivity rate was significantly lower in PD-L1-positive HRS cases than PD-L1-negative HRS cases when 9A11 and SP142 were used for staining (11.5% (3/26) vs. 39.3% (11/28), *P*=0.020; 12.5% (4/32) vs. 45.5% (10/22) *P*=0.011). However, no such difference was found when 22C3 was used (*P*=0.337). When IC PD-L1 expression was considered, CD163+ TAM density (%) showed the highest correlation with PD-L1 expression (moderate concordance) among all subtypes of ICs, especially when 9A11 was used for staining (kappa index 0.69); the details are shown in Fig. 5.

### 9A11 staining to determine PD-L1 expression status and its associations with clinicopathological features

Overall, PD-L1 immunostaining with 9A11 showed the following results: 87.0% (47/54) of cases were found to have a high level of PD-L1 expression: 48.1% (26/54) were evaluated as having PD-L1 positivity for HRS cells, and 85.2% (46/54) were evaluated as having PD-L1 positivity for ICs. The expression of PD-L1 in either HRS cells or ICs showed poor concordance with other clinical factors (including sex, age, morphological subtype, EBER status, and stage). The results are further analysed and shown in Fig. S3A and S3B.

## Discussions

We chose 3 representative PD-L1 antibody clones for IHC assay comparisons: 405.9A11 (9A11) was first used by Ansell et al. [8] in their outstanding study in CHL. 22C3 is considered a sensitive antibody [39]; it was the first FDA-approved Ab and is the most widely used Ab recently [18]. SP142 was the 1st clinically validated Ab for both TC and IC [38, 42]. The RNAscope assay, which is an antibody-independent assay that employs FFPE samples, was recently developed to detect PD-L1 expression at the mRNA level; it has been applied in breast, lung, and gastric tumours [23–25]. The specificity, reproducibility, and objectivity of RNAscope compared to IHC have been reported in gastric cancer [24, 25]. Because both HRS cells and reactive ICs in CHL can express PD-L1, mainly in a membranous pattern, the PD-L1-positive ICs can be so tightly packed around the HRS cells that it is difficult to tell whether the HRS cells express PD-L1 (as shown in Fig. 3A). In this situation, detection of a “dot-like” pattern of PD-L1 mRNA in the cytoplasm of HRS cells by RNAscope is much easier and provides better information for evaluation (Fig. 3C). The RNAscope assay was successfully applied to assess our CHL cases, and a provisional scoring system was also developed.

We showed that IHC using 9A11 provided the most accurate results, showing a nearly perfect correlation with the RNAscope assay. 9A11 is an anti-PD-L1 Ab clone that binds the cytoplasmic domain and thus is more selective for membranous PD-L1; it has shown stronger staining and more membrane and less cytoplasmic staining [50], which makes it easier for 9A11 to distinguish the membranous staining of HRS cells from that of surrounding ICs, resulting in higher staining intensity and specificity (shown in Fig. 3A).

Regarding PD-L1 expression in HRS cells, nearly half of the cases expressed high levels of protein or mRNA (48.1% of cases by IHC with clone 9A11 and 45.7% of cases by RNAscope), in line with the recently published results from Veldman et al. [16], although much lower than the 100% positivity rate of R/R cases reported by Ansell [8]. In the study from Roemer et al. [12], alterations of the 9p24.1 gene encoding PD-L1 in HRS cells in CHL included copy gain (56%) and amplification (36%) alterations, and patients with amplifications were found to have significantly increased PD-L1 expression and shorter PFS [12]. However, only 4 out of 10 cases were found to have gene amplification in the R/R CHL study of Ansell [8]. The discordance in the PD-L1-positive rate might be caused by the different evaluation criteria used among researchers.

Study of Veldman et al. found that 69% of CHL cases had positive PD-L1 expression on ICs based on an assay with the Ab clone E1L3N [16]. In our study, we found that IC PD-L1-positive rates were 85.2% (9A11), 68.5% (SP142), and 74.1% (22C3), and PD-L1 was most likely to be expressed by TAMs, as proven by statistical analysis. The majority of CHL cases express high levels of PD-L1 on ICs; however, it should be noted that different antibody clones will provide different positive rates. 9A11 was the most sensitive and SP142 had the lowest sensitivity in a previous lung cancer study by Tsao et al. [39].

In our study, PD-L1 expression, either on tumoral HRS cells or on ICs, showed poor concordance with clinicopathological factors, including sex, age, morphological subtype, and EBER status. Green et al. [1] reported that EBV-positive CHL has upregulated PD-L1 expression [1, 43]. However, Paydas et al. did not find this association [51], and neither did our study. Since the high level of PD-L1 expression is so prevalent in CHL it could not be caused solely by EBV.

Many subtypes T cells and TAMs in the microenvironment were subclassified by IHC marker assessment of our series of CHL cases. However, HRS cell PD-L1 expression status (by 9A11 assay) correlated poorly with Th (CD4+), Treg (FOXP3+), CTL (CD8+), and TAM (CD163+) density. Increased PD-L1 expression on ICs correlated best with a higher density of CD163+ TAMs. The same finding was seen in a lung cancer study [32]. The percentage of PD-L1-positive ICs was significantly higher in the TAM-high group than in the TAM-low group. In addition, PD-L1 expression on TAMs may be derived from HRS cells [27]. There are controversial results regarding PD-1+ cell densities [9], which range from low [52] to very high [53]. In our study, only approximately 1/4 of cases presented with a “high” level of PD-1-positive cells (cut-off value ≥10%). In those cases, with a high level of PD-L1 on HRS cells, there was a significantly lower rate of PD-1 positivity; this reverse correlation may exist to prevent overactivation of the PD-1 pathway and maintain the immunosuppressive balance in CHL. Although various numbers of FOXP3+ Tregs have been reported previously [54, 55], different from other tumours [30, 31, 56], the number of FOXP3+ Tregs was not associated with the PD-L1 expression level (in either HRS cells or ICs) in our series.

An increased number of tumour-associated macrophages [45], especially higher density (>25%) CD163-positive TAMs (represent M2 macrophages) [33]has been found to be strongly associated with shortened survival. High level of PD-L1 Expression was also an adverse predictor of clinical outcome in a previous report. and in various tumours, including diffuse large B-cell lymphoma [31, 48, 57]. In agreement with the strongest relationship being between CD163-positive TAM density and PD-L1 expression level, patients with higher levels of PD-L1 in ICs had a worse OS (see in supplementary materials).

## Conclusions

405.9A11 provided the most convincing results for evaluating the expression of PD-L1 in HRS cells, as proven independently by comparisons with the results of the RNAscope assay, and was also the most sensitive for detecting PD-L1 expression in ICs. A high level of PD-L1 expression was prevalent in CHL, and high PD-L1 expression was more frequent in ICs than in HRS cells. Thus, pathologists should report PD-L1 expression in a combined manner, including both the positive rate of HRS cells and the positive percentage of ICs.

### Supplementary Information

Figure S1A **Analysis of relative densities of different types of background immune cells by immunostaining with subgroup associated markers in CHL patients.** Picture A-E subsequently shew the representative high-power fields with high-density for CD4 (marking T-helper cells), FOXP3(marking T-reg cells), CD8 (marking cytotoxic T-cells), CD163(marking macrophages) and PD1 positive T helper cells from relative CHL patients. In contrast，Picture F-J subsequently shew the representative high-power fields of CHL cases with low-density for CD4, FOXP3, CD8, CD163 and PD1. (PNG 6476 kb)

High Resolution Image (TIF 10237 kb)

Figure S1B (PNG 6340 kb)

High Resolution Image (TIF 10237 kb)

Figure S1C (PNG 6664 kb)

High Resolution Image (TIF 10237 kb)

Figure S1D (PNG 6186 kb)

High Resolution Image (TIF 10237 kb)

Figure S1E (PNG 6306 kb)

High Resolution Image (TIF 10237 kb)

Figure S1F (PNG 6883 kb)

High Resolution Image (TIF 10237 kb)

Figure S1G (PNG 4753 kb)

High Resolution Image (TIF 10237 kb)

Figure S1H (PNG 5886 kb)

High Resolution Image (TIF 10237 kb)

Figure S1I (PNG 6068 kb)

High Resolution Image (TIF 10237 kb)

Figure S1J (PNG 5574 kb)

High Resolution Image (TIF 10237 kb)

Figure S2 **Correlations between PD-L1 expression on HRS cells and different subgroups of immune cells in TME.** We use the of PDL 1 expression status detected with 9A11 clone Abs and for IHC Sore for the densities of different subgroups of immune cells for statistical analysis. (PNG 187 kb)

High Resolution Image (TIF 95 kb)

Figure S3A and Figure S3B  **Correlations between the PD-L1 expression clinicopathological parameters.** Statistically analysis was done for correlation between PDL1 expression on CHL cells (HRS cells /background IC cells) and the clinicopathological parameters (sex, age, B symptoms, stage, pathology, EBER et al), detected by IHC with 9A11 clone Abs (JPG 70 kb)

Figure S4A **Overall survival and event free survival analysis for the impact of CD163+ tumor associated macrophages and PD-L1 expression on IC**. CHL cases were grouped according to **CD163+ tumor associated macrophages** density, as 1+:<5%, 2+:5-25%，and 3+: >25%) in Figure S4A and4B; and was groups according to PD-L1 expression level on immune cells (ICs) with 9A11 assay in Figure S4C and 4D, the cut off value as >25% named “high-IC”, or else “low-IC”), All were with univariable survival analysis, Kaplan-Meier method. (JPG 27 kb)

Figure S4B (PNG 62 kb)

High Resolution Image (TIF 50 kb)

Figure S4C (JPG 41 kb)

Figure S4D (PNG 96 kb)

High Resolution Image (TIF 102 kb)

Supplementary table 1 (DOCX 16 kb)

Supplementary material-IHC methology&suvival analysis (DOCX 28 kb)

## Data Availability

All data generated or analysed during this study are included in this published article (and its supplementary information files).
