# Supplementary figures and images for: PD-L1 immunohistochemistry assay optimization to provide more comprehensive pathological information in classic Hodgkin lymphoma
Source: J Hematop. 2023 Feb 1;16(1):7–16. doi: 10.1007/s12308-023-00530-1 (PMC10766715; doi:10.1007/s12308-023-00530-1)

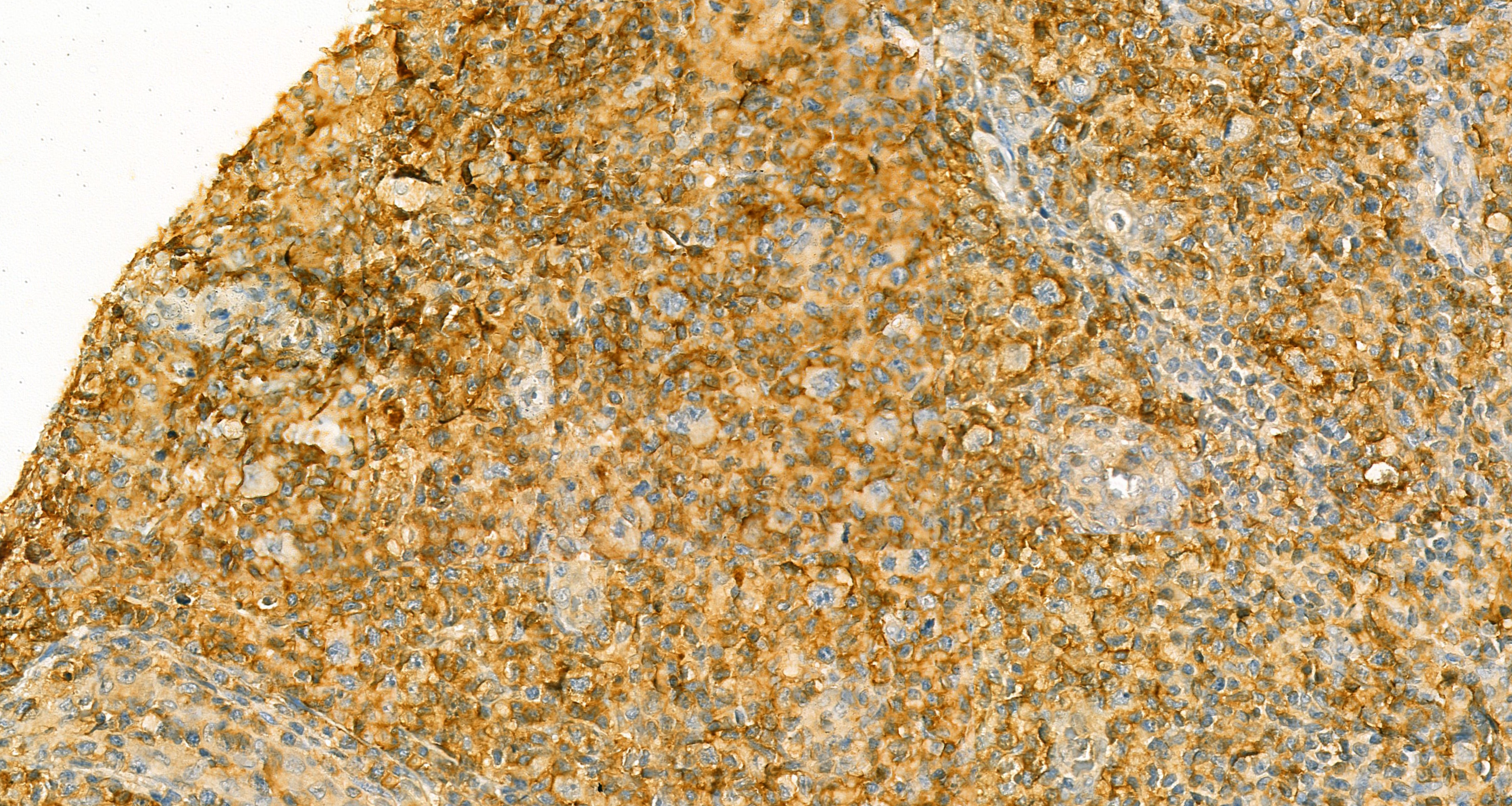

Supplement: Supplementary file 1 — Analysis of relative densities of different types of background immune cells by immunostaining with subgroup associated markers in CHL patients. Picture A-E subsequently shew the representative high-power fields with high-density for CD4 (marking T-helper cells), FOXP3(marking T-reg cells), CD8 (marking cytotoxic T-cells), CD163(marking macrophages) and PD1 positive T helper cells from relative CHL patients. In contrast，Picture F-J subsequently shew the representative high-power fields of CHL cases with low-density for CD4, FOXP3, CD8, CD163 and PD1. (PNG 6476 kb) [file 12308_2023_530_Fig6_ESM.png]

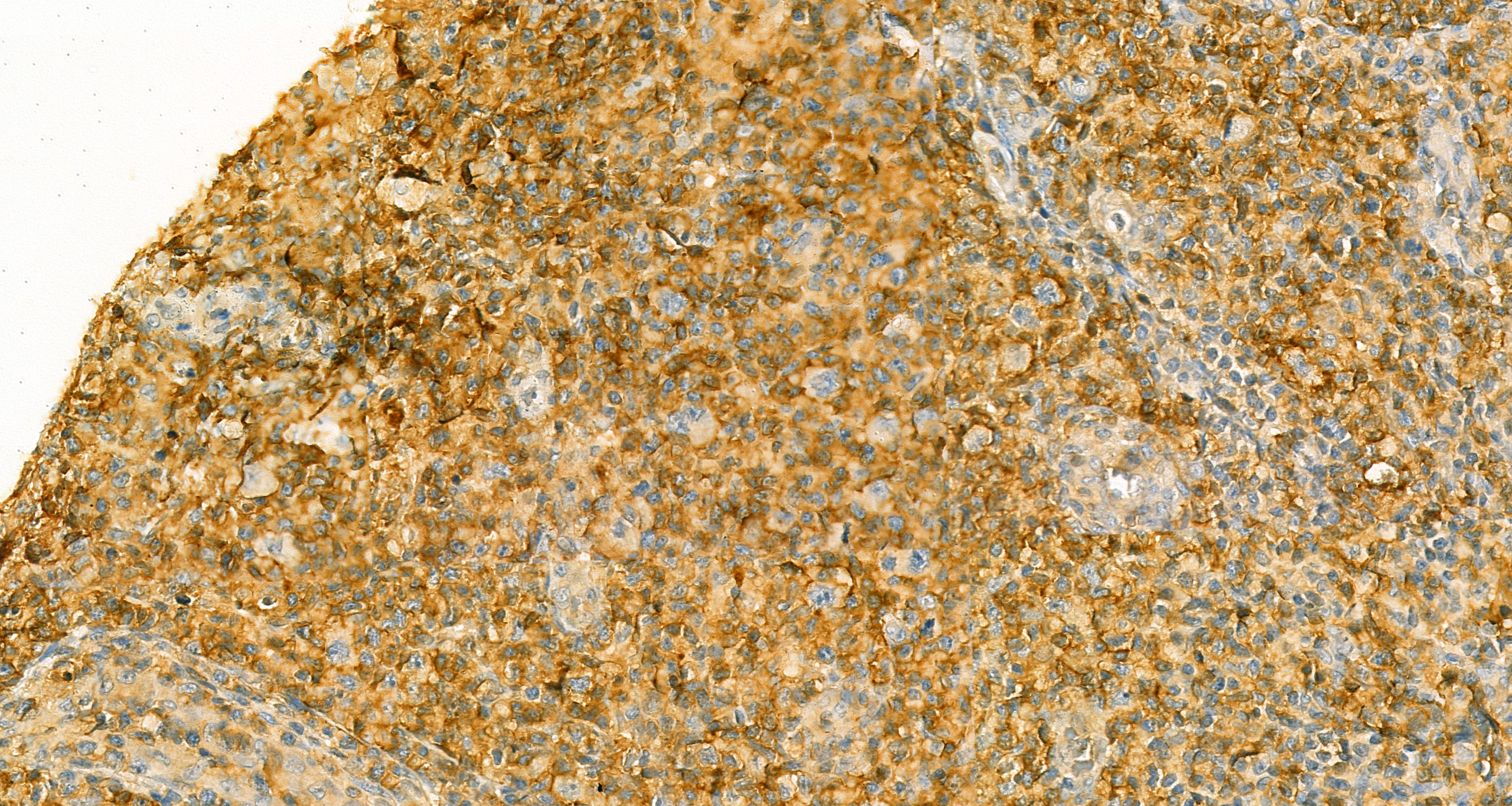

Supplement: Supplementary file 2 — High Resolution Image (TIF 10237 kb) [file 12308_2023_530_MOESM1_ESM.tif]

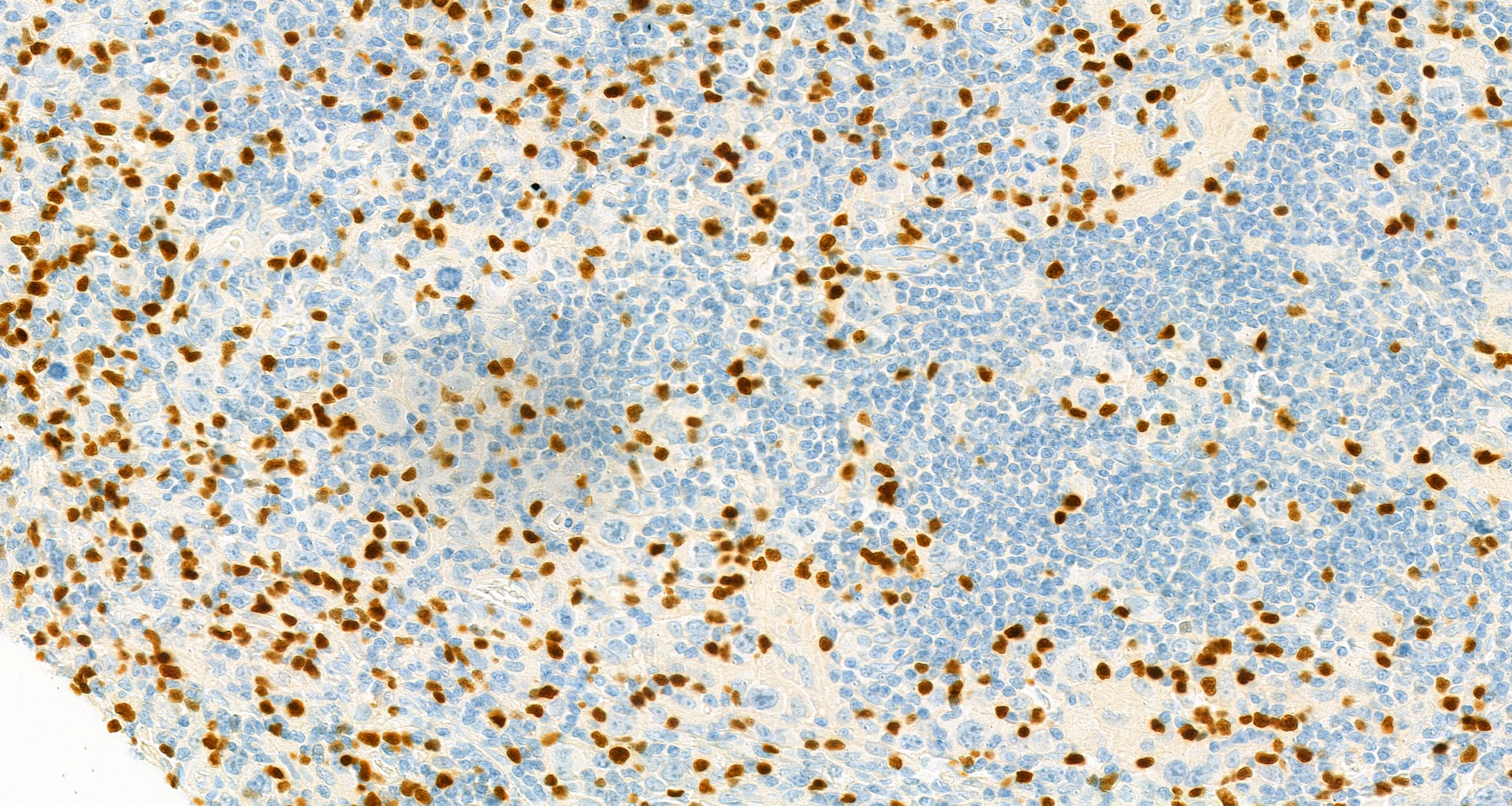

Supplement: Supplementary file 3 — (PNG 6340 kb) [file 12308_2023_530_Fig7_ESM.png]

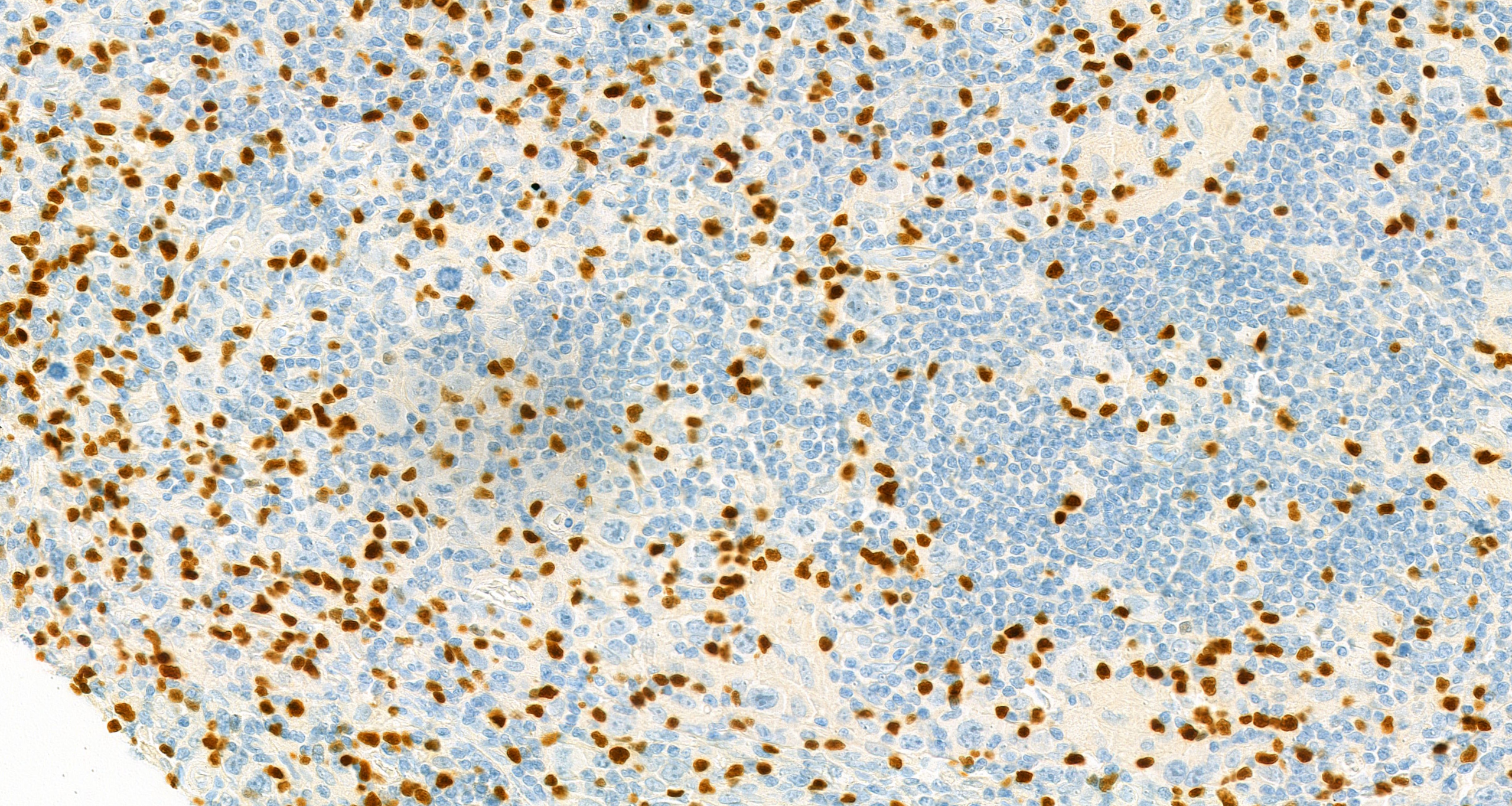

Supplement: Supplementary file 4 — High Resolution Image (TIF 10237 kb) [file 12308_2023_530_MOESM2_ESM.tif]

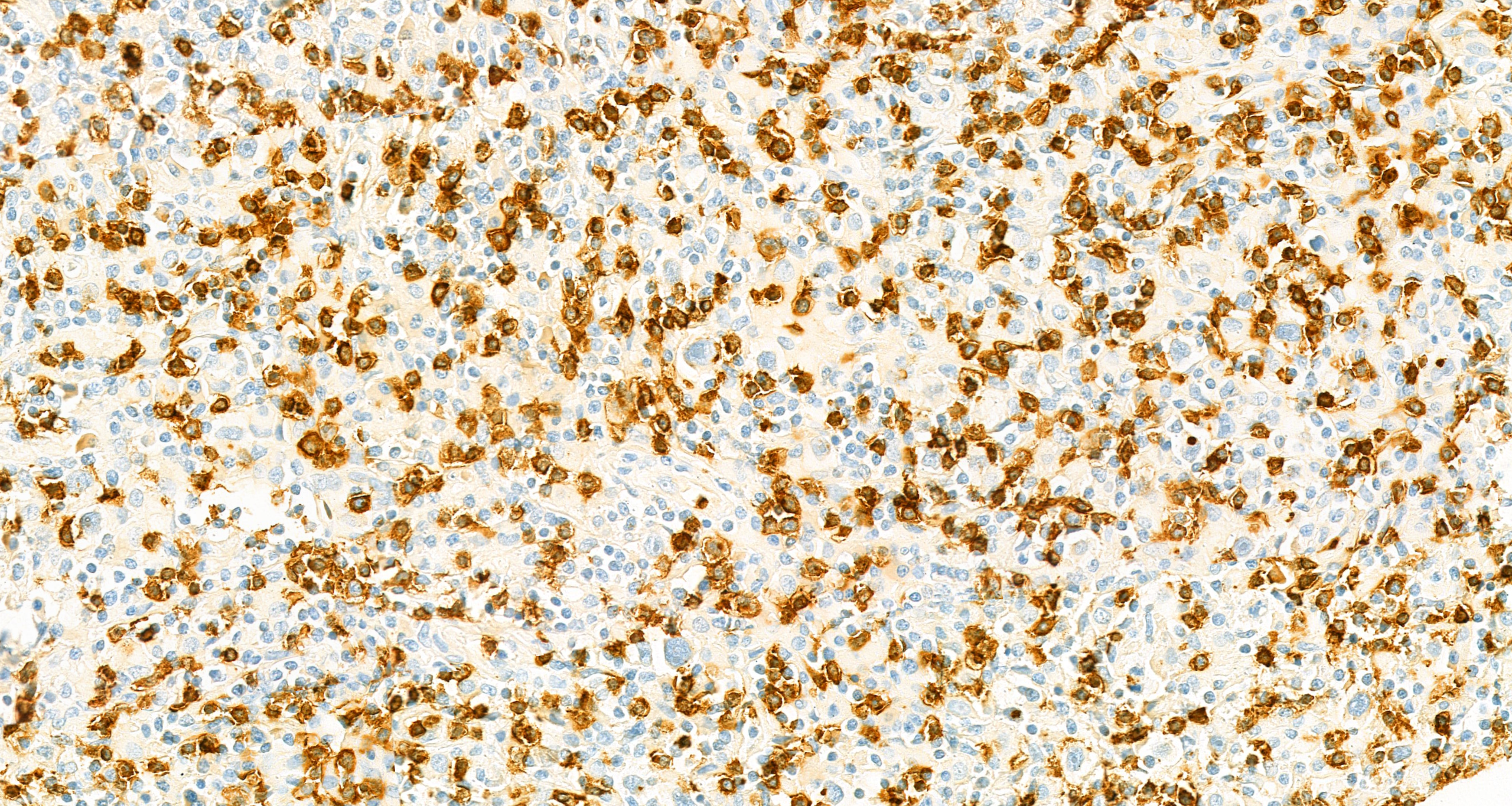

Supplement: Supplementary file 5 — (PNG 6664 kb) [file 12308_2023_530_Fig8_ESM.png]

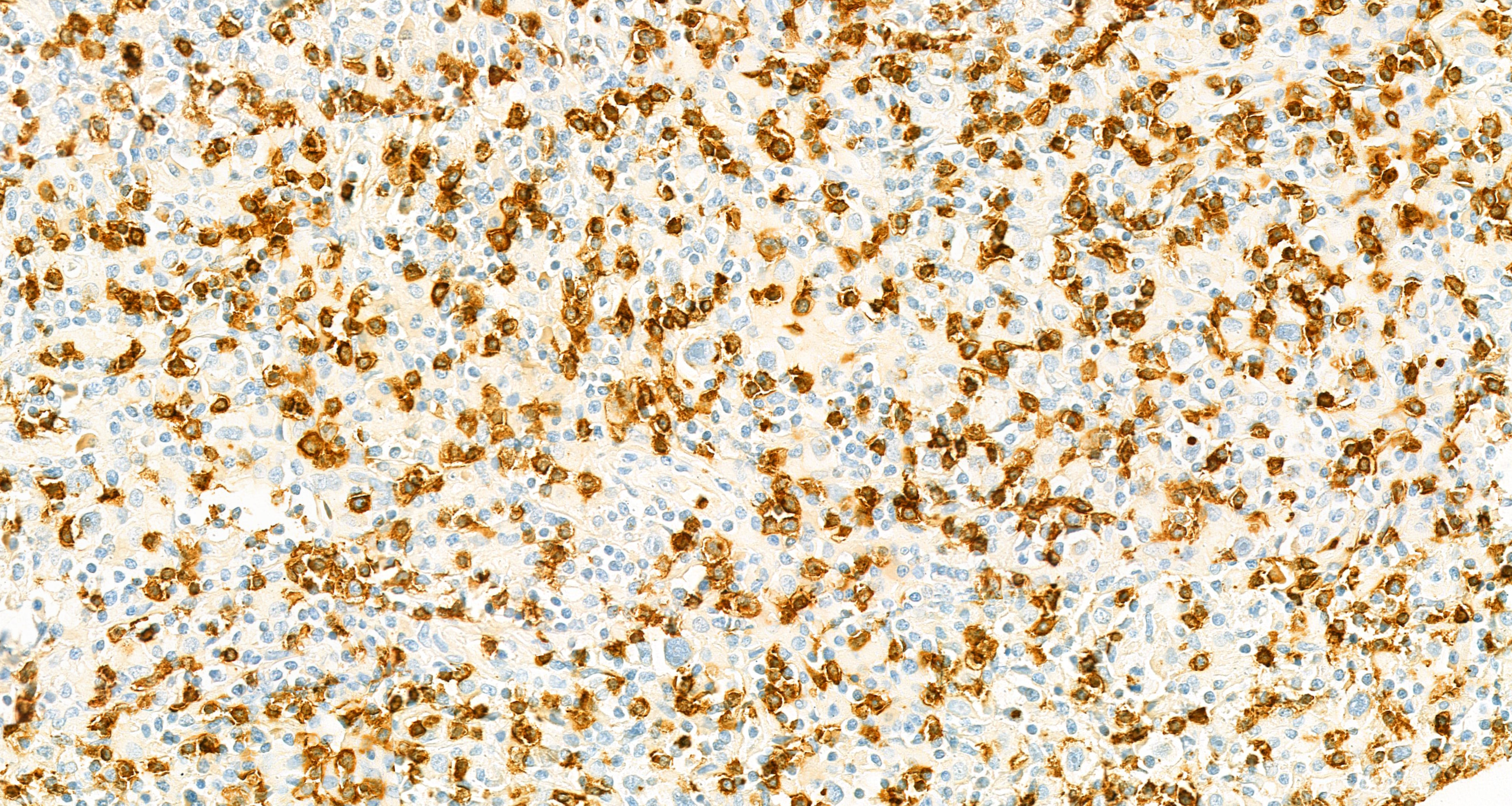

Supplement: Supplementary file 6 — High Resolution Image (TIF 10237 kb) [file 12308_2023_530_MOESM3_ESM.tif]

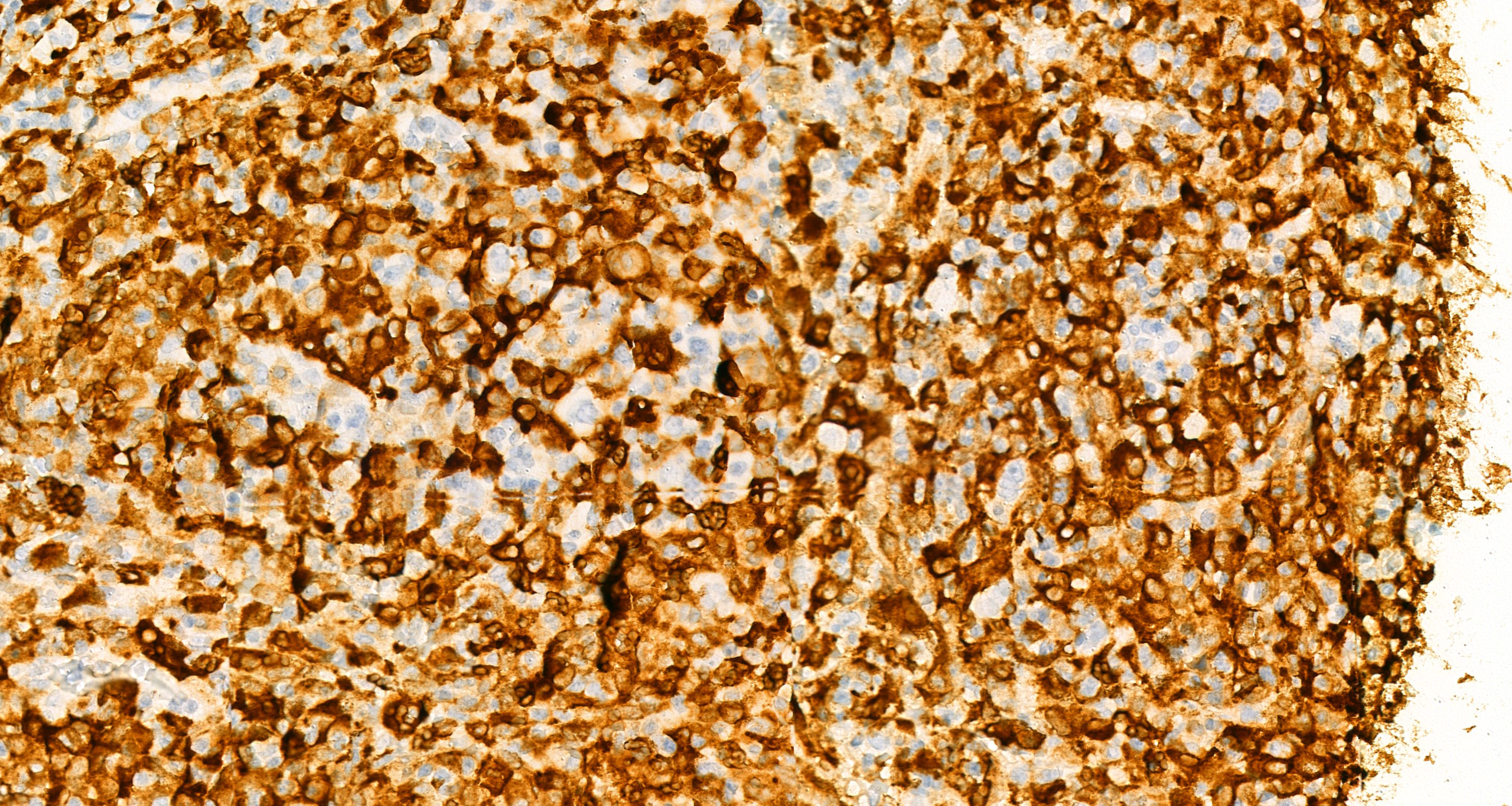

Supplement: Supplementary file 7 — (PNG 6186 kb) [file 12308_2023_530_Fig9_ESM.png]

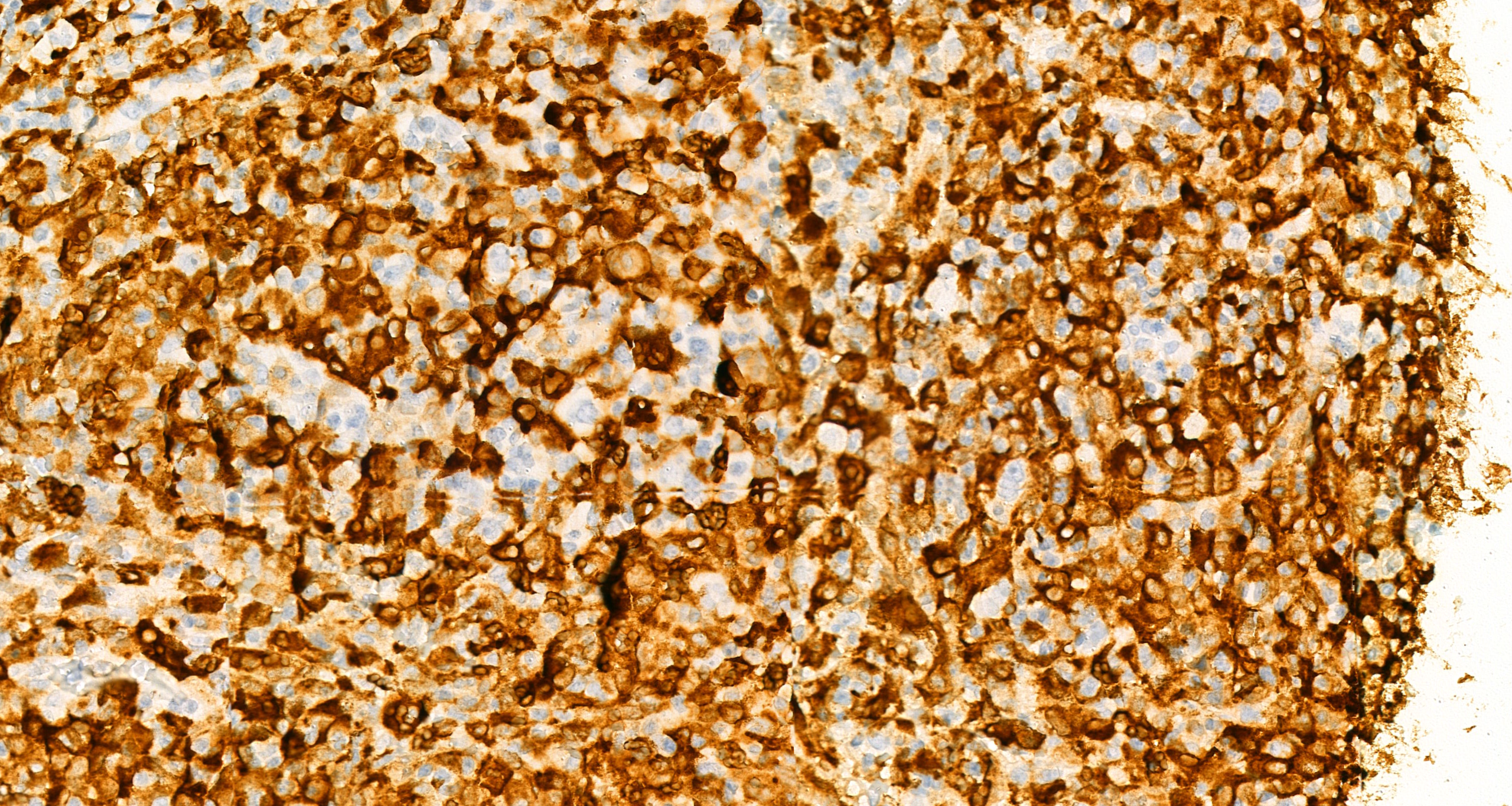

Supplement: Supplementary file 8 — High Resolution Image (TIF 10237 kb) [file 12308_2023_530_MOESM4_ESM.tif]

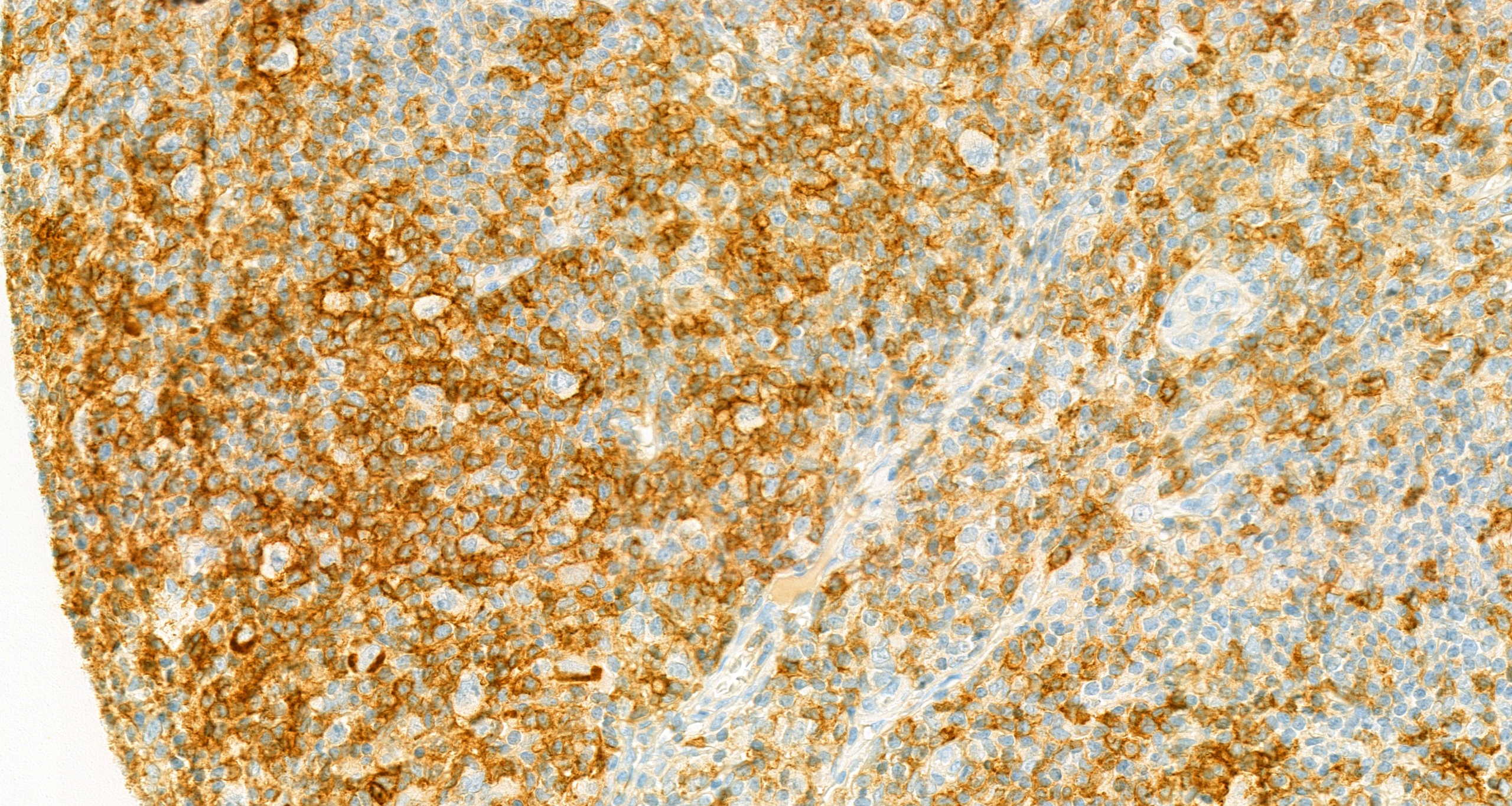

Supplement: Supplementary file 9 — (PNG 6306 kb) [file 12308_2023_530_Fig10_ESM.png]

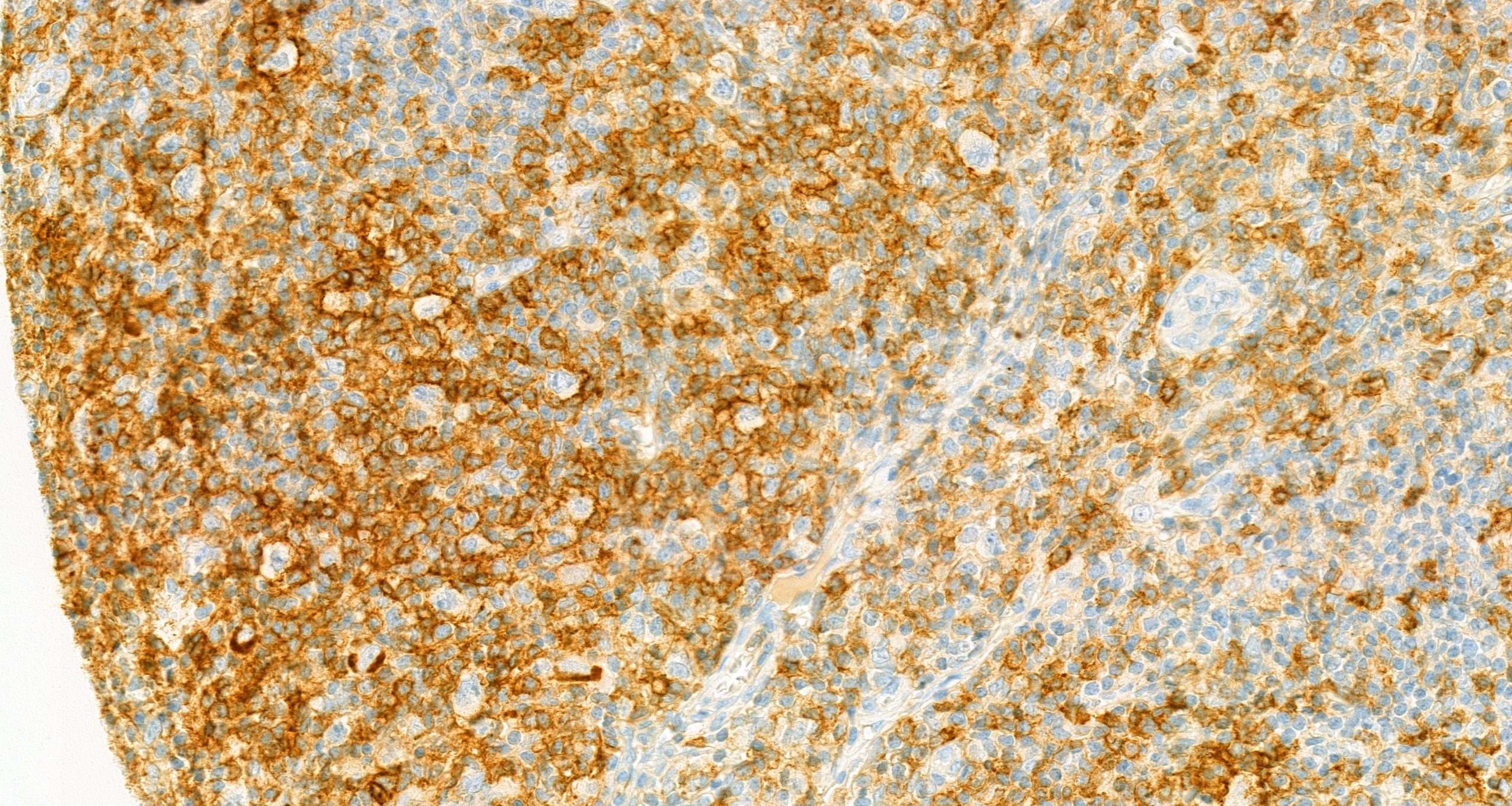

Supplement: Supplementary file 10 — High Resolution Image (TIF 10237 kb) [file 12308_2023_530_MOESM5_ESM.tif]

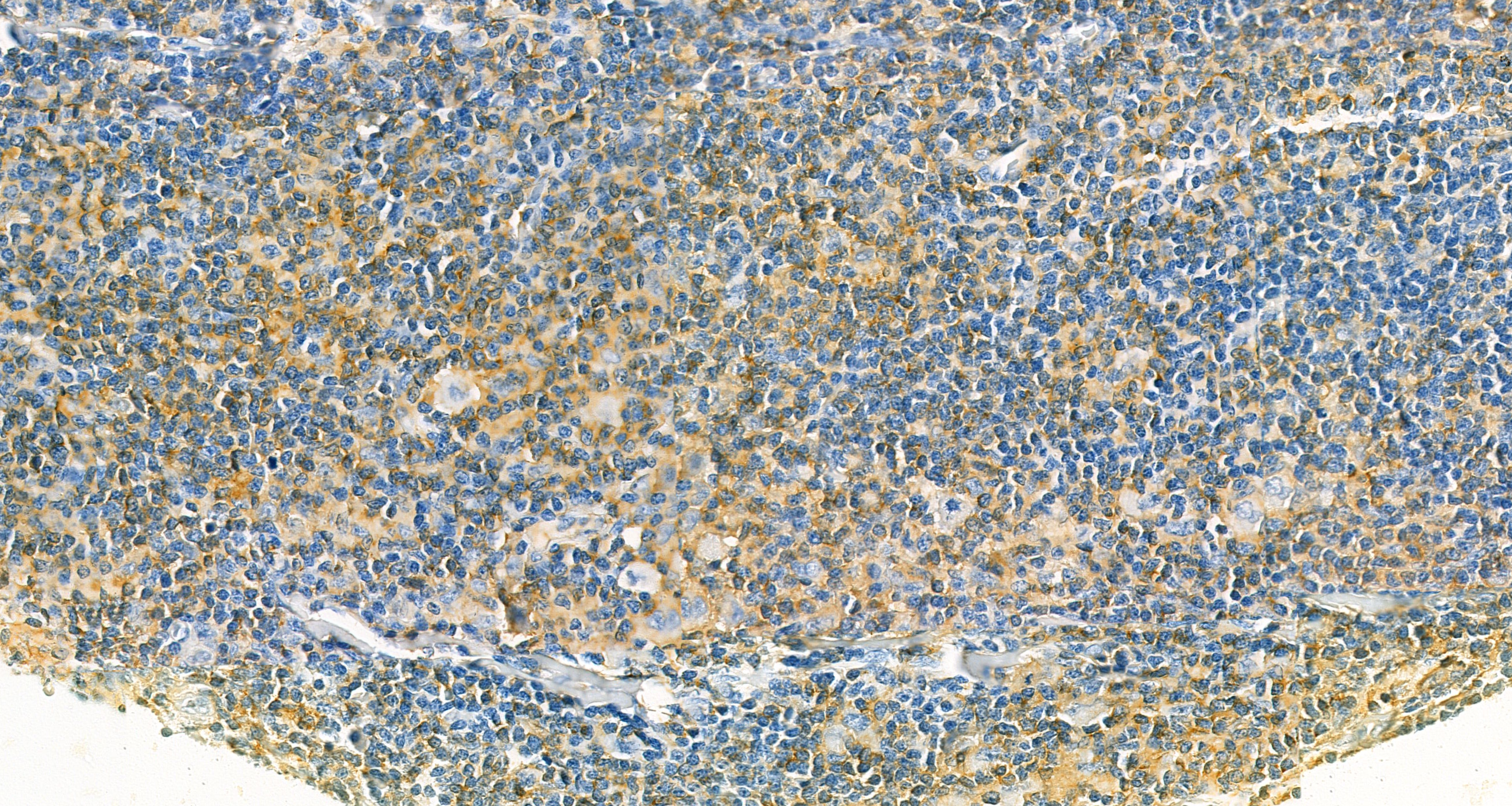

Supplement: Supplementary file 11 — (PNG 6883 kb) [file 12308_2023_530_Fig11_ESM.png]

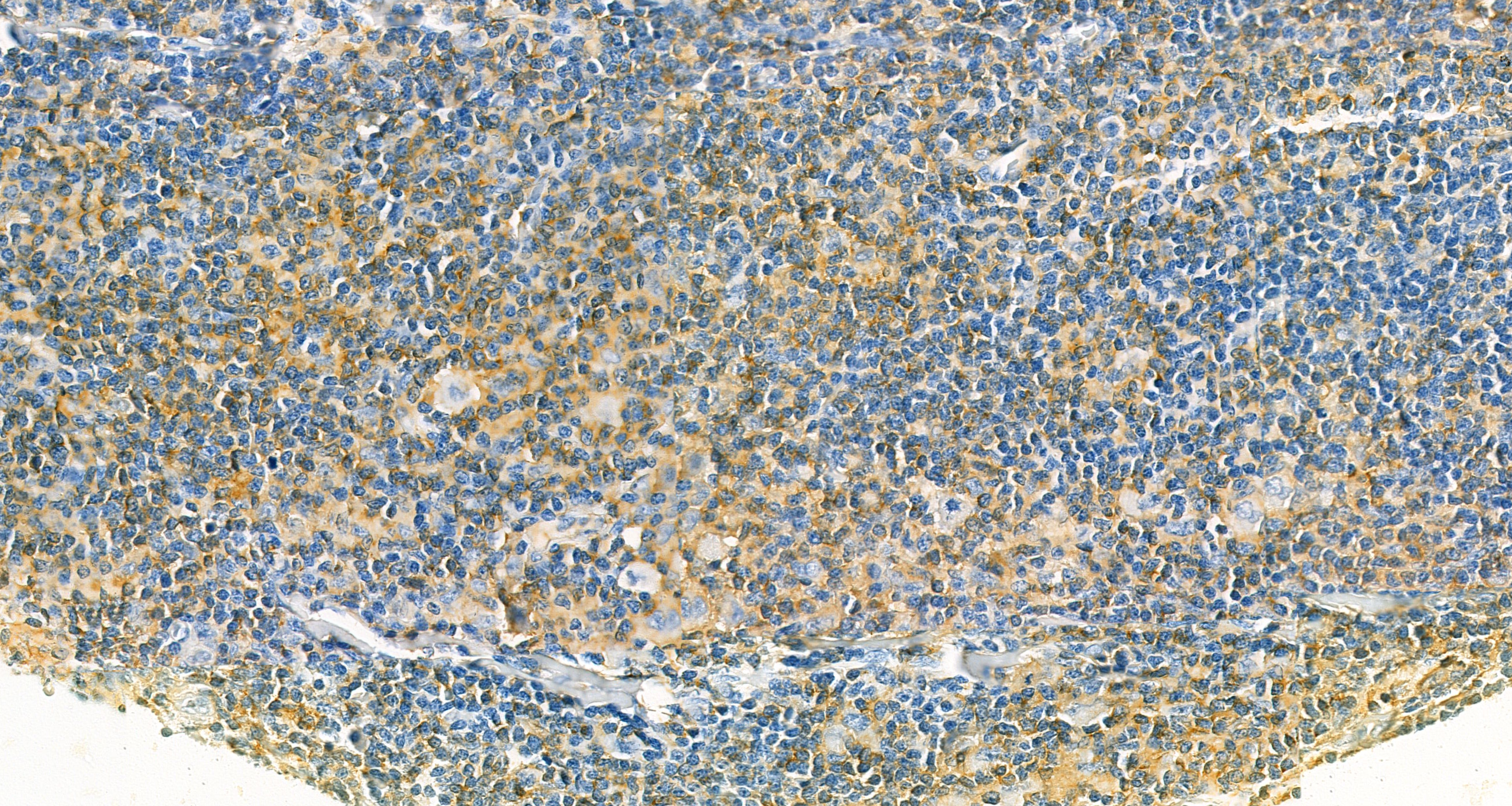

Supplement: Supplementary file 12 — High Resolution Image (TIF 10237 kb) [file 12308_2023_530_MOESM6_ESM.tif]

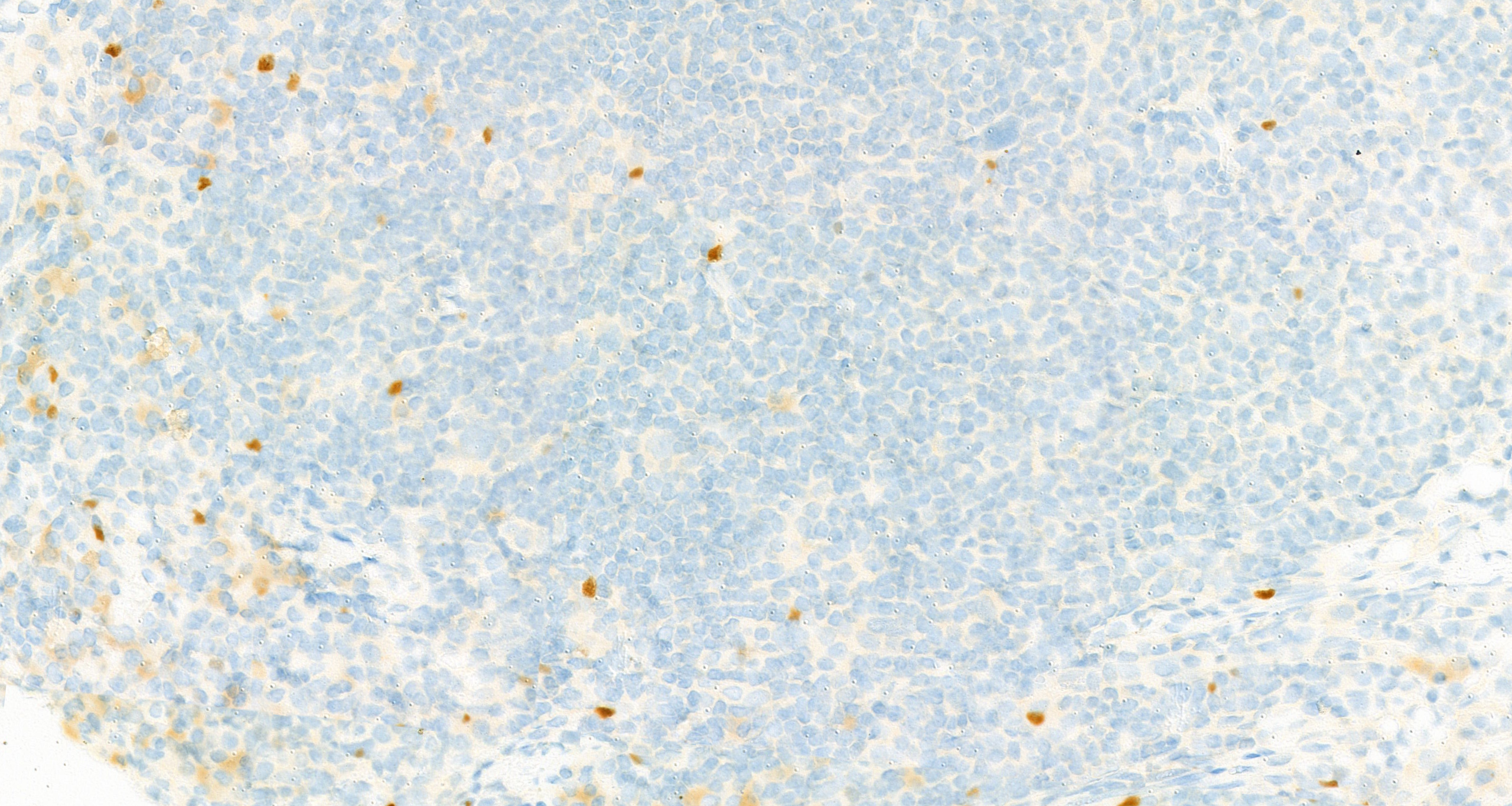

Supplement: Supplementary file 13 — (PNG 4753 kb) [file 12308_2023_530_Fig12_ESM.png]

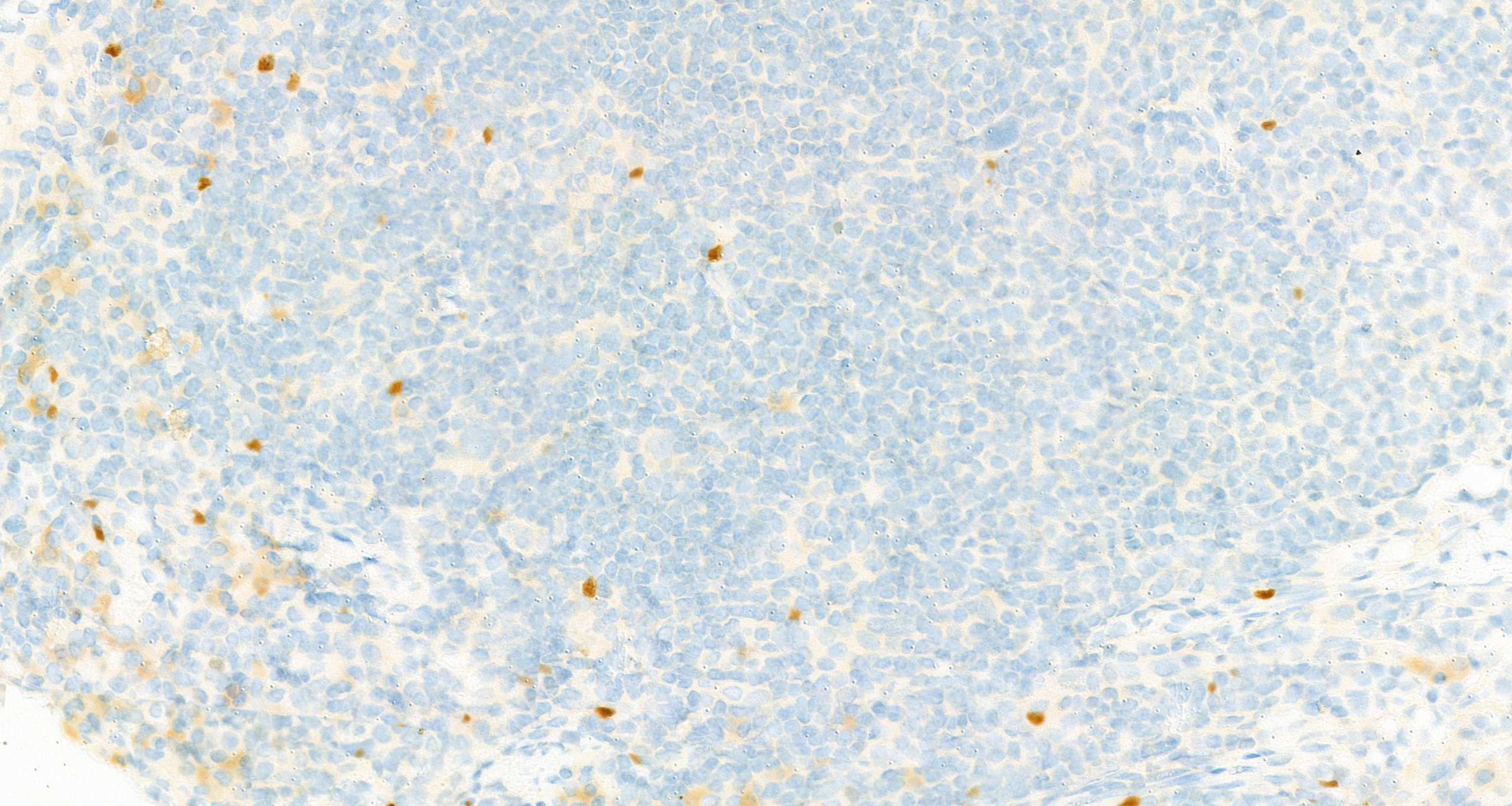

Supplement: Supplementary file 14 — High Resolution Image (TIF 10237 kb) [file 12308_2023_530_MOESM7_ESM.tif]

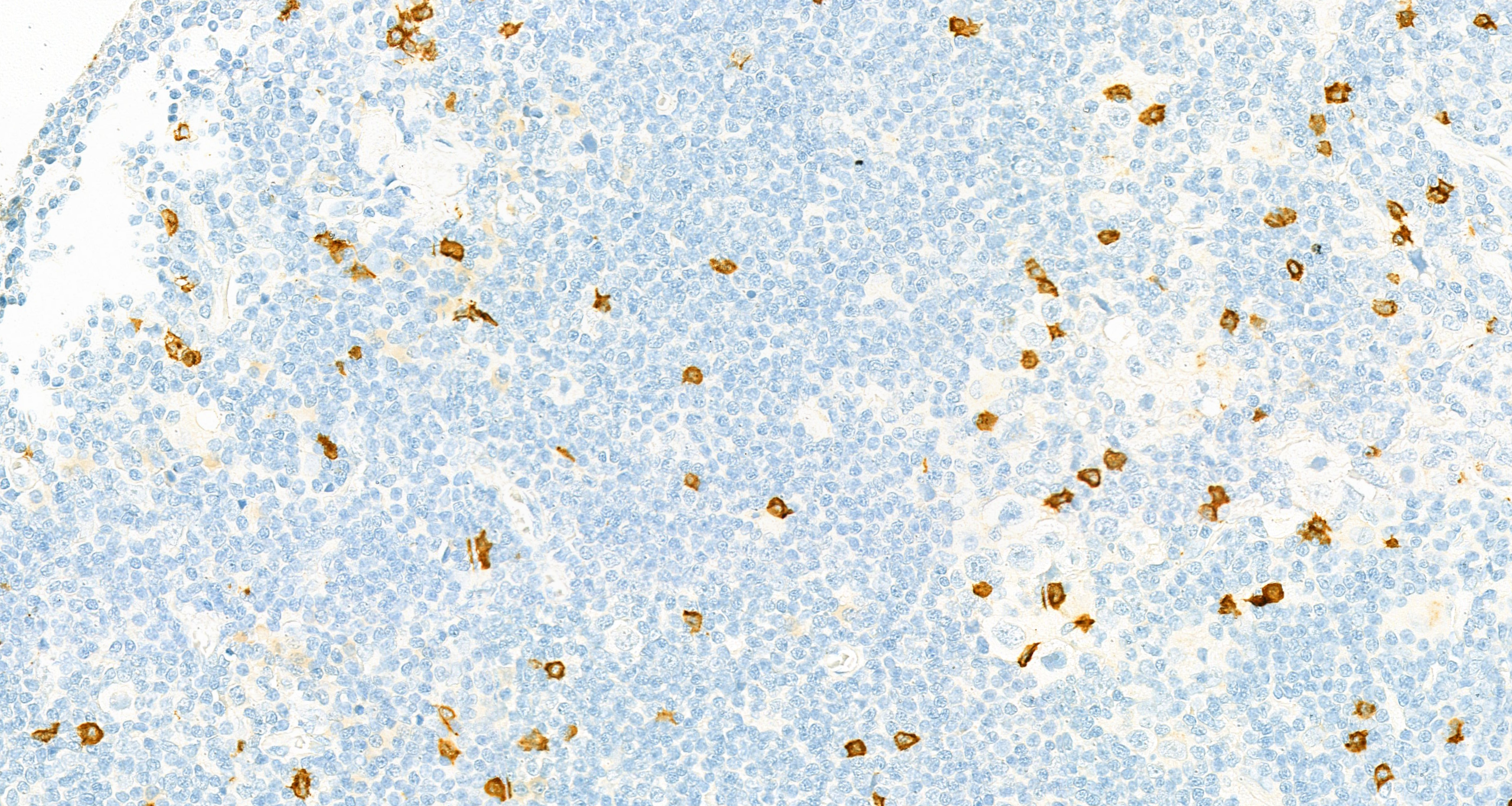

Supplement: Supplementary file 15 — (PNG 5886 kb) [file 12308_2023_530_Fig13_ESM.png]

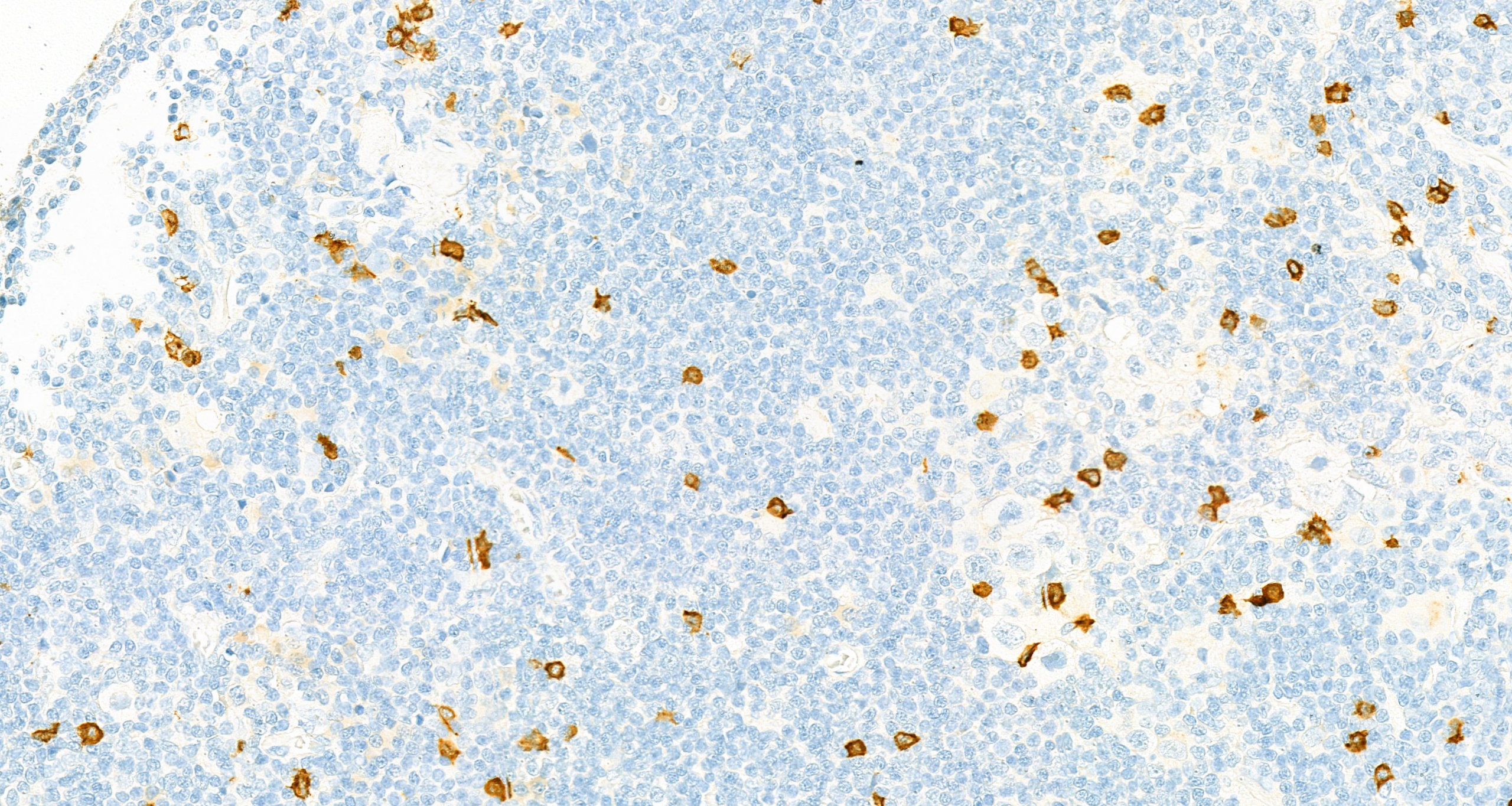

Supplement: Supplementary file 16 — High Resolution Image (TIF 10237 kb) [file 12308_2023_530_MOESM8_ESM.tif]

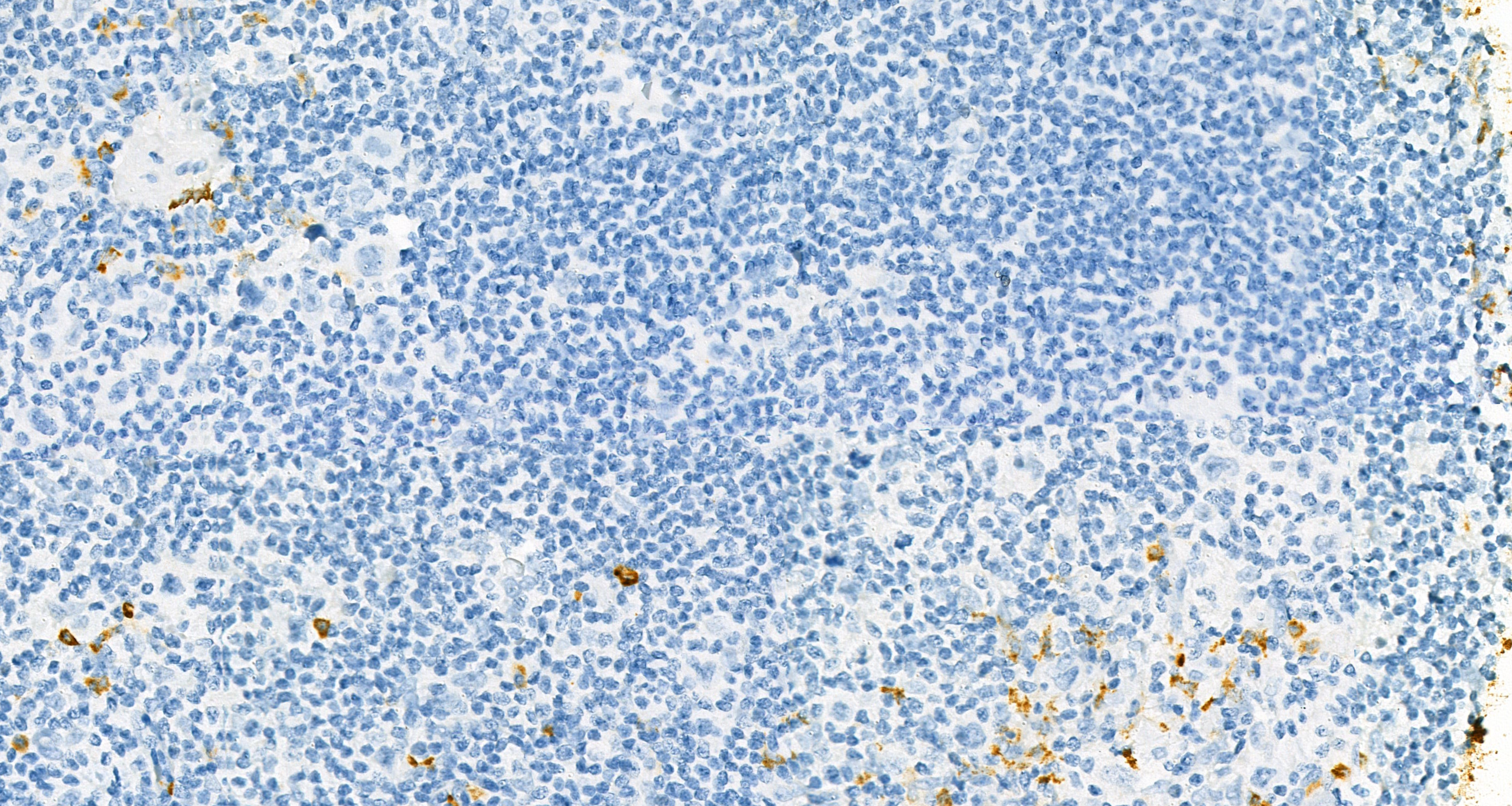

Supplement: Supplementary file 17 — (PNG 6068 kb) [file 12308_2023_530_Fig14_ESM.png]

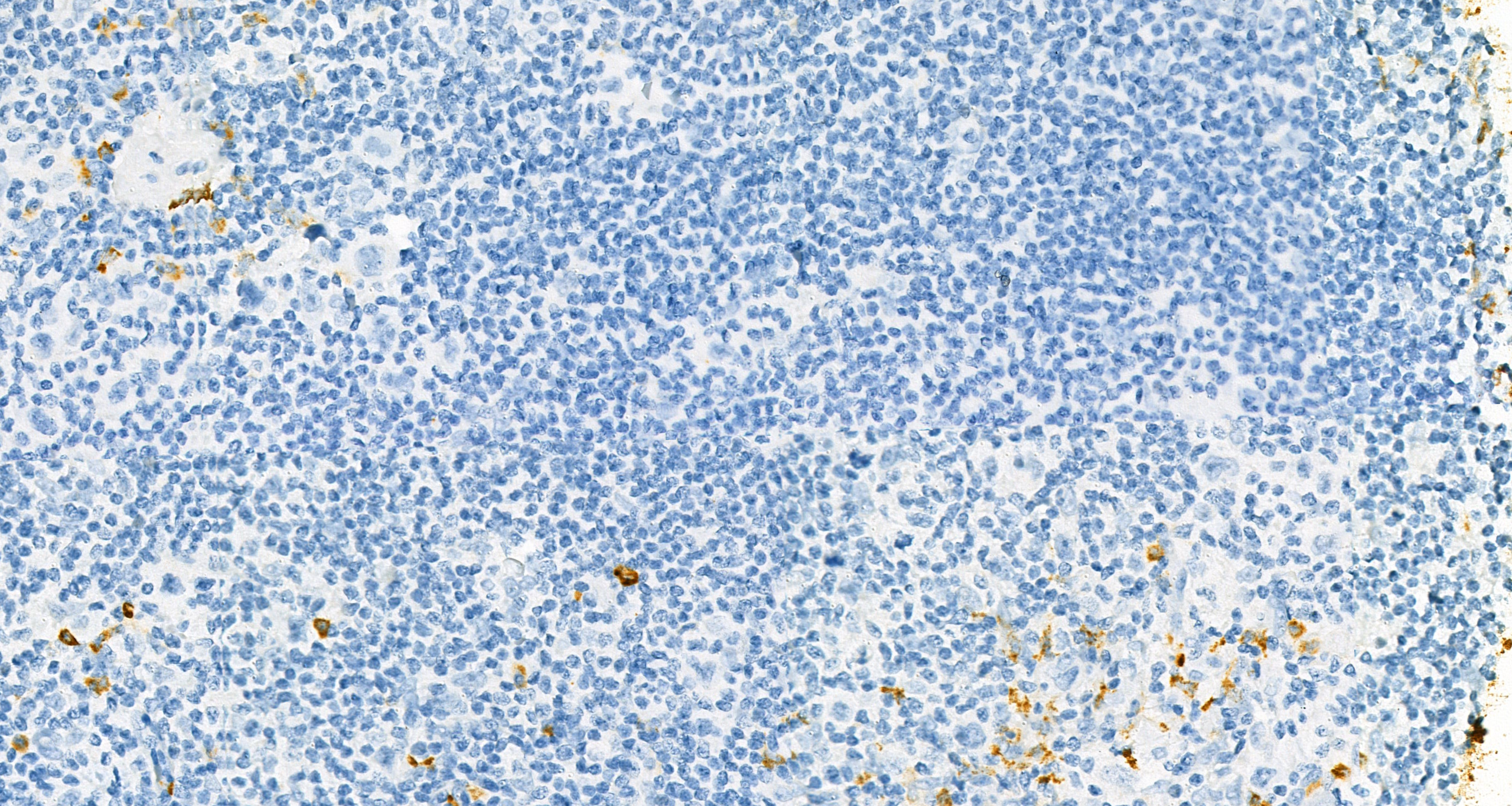

Supplement: Supplementary file 18 — High Resolution Image (TIF 10237 kb) [file 12308_2023_530_MOESM9_ESM.tif]

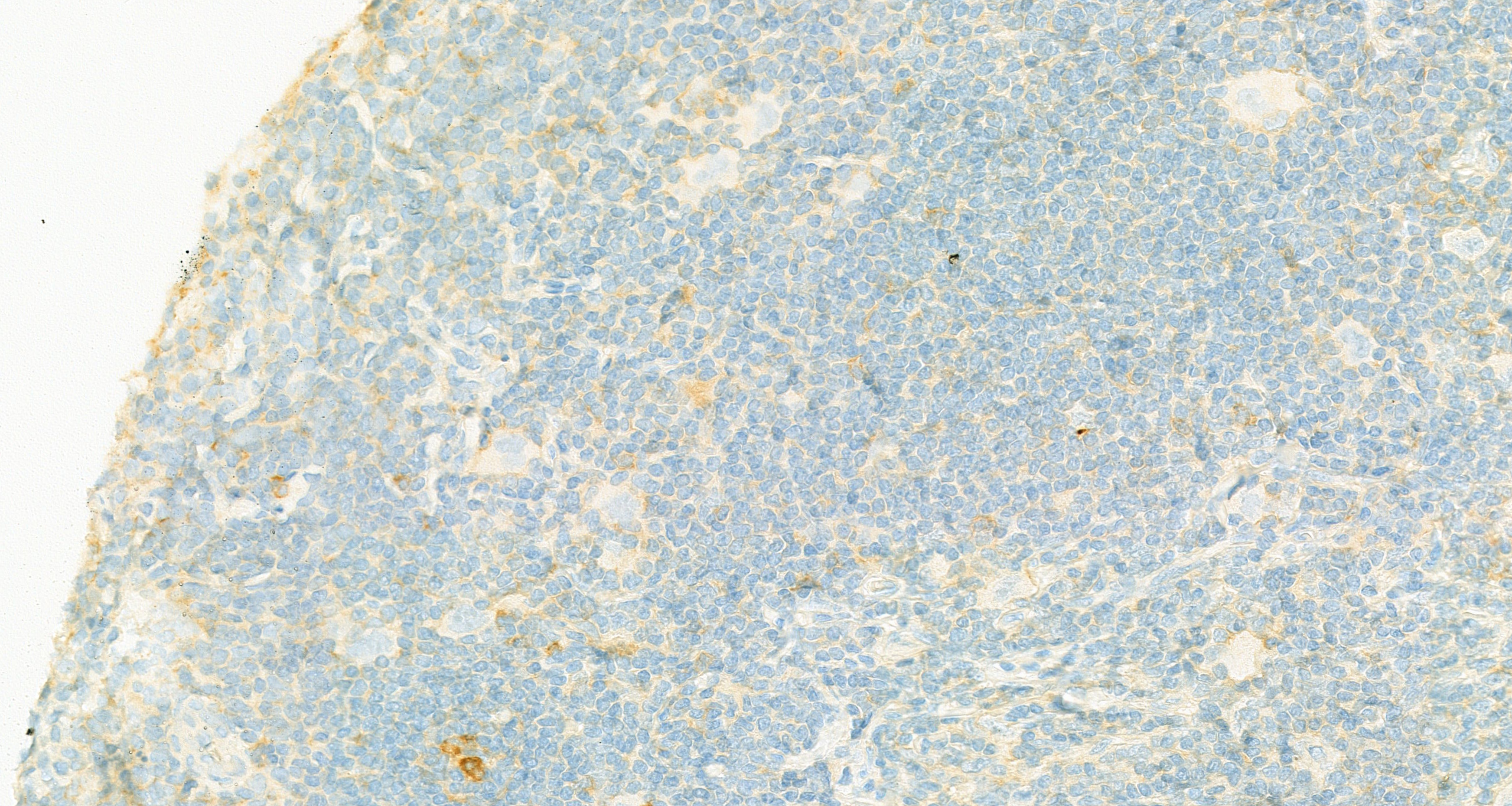

Supplement: Supplementary file 19 — (PNG 5574 kb) [file 12308_2023_530_Fig15_ESM.png]

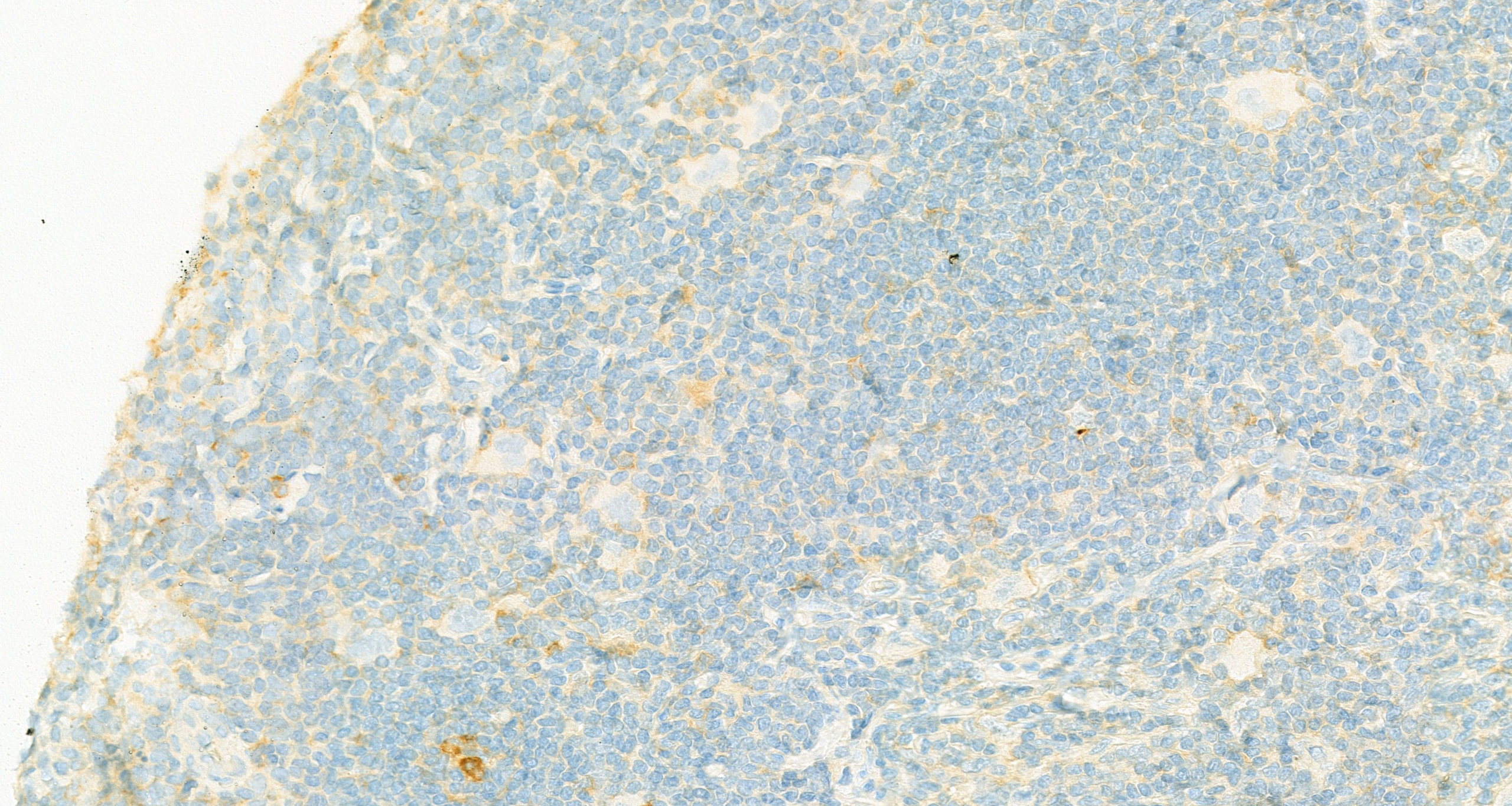

Supplement: Supplementary file 20 — High Resolution Image (TIF 10237 kb) [file 12308_2023_530_MOESM10_ESM.tif]

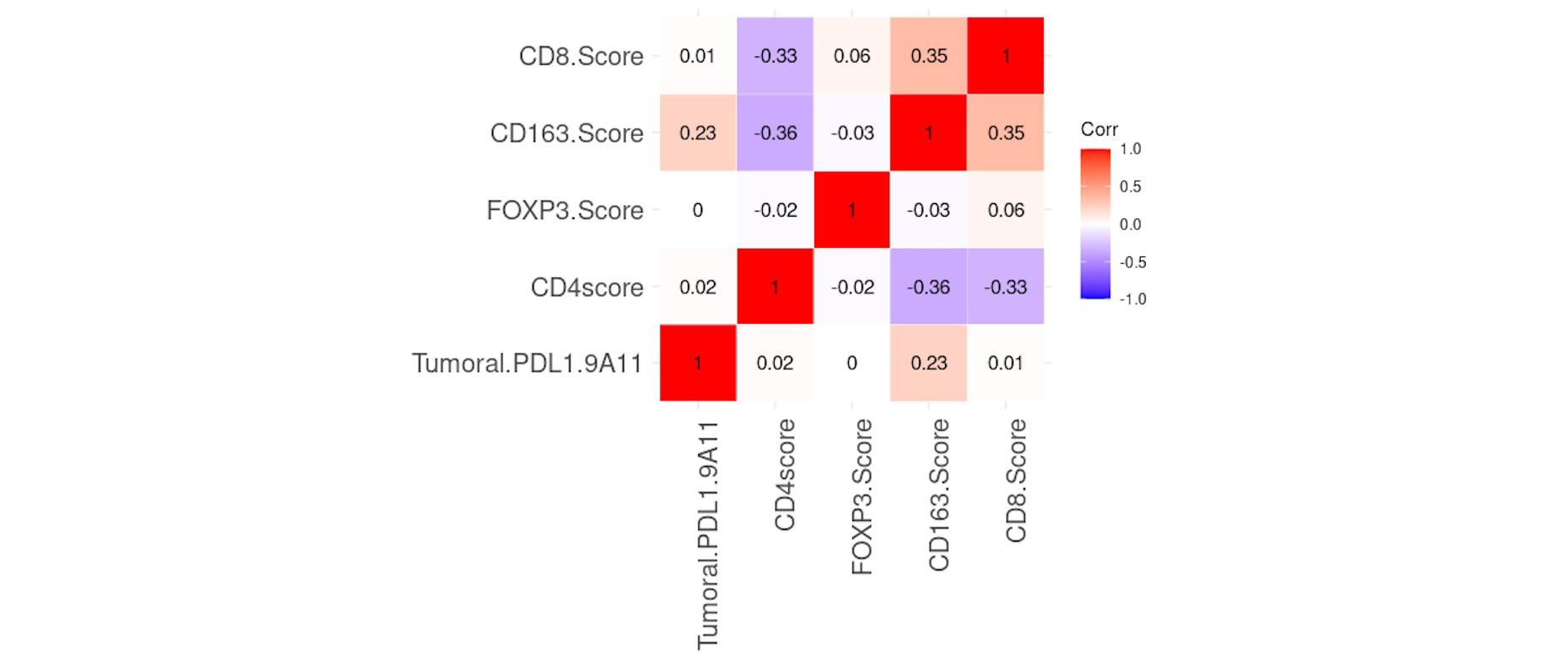

Supplement: Supplementary file 21 — Correlations between PD-L1 expression on HRS cells and different subgroups of immune cells in TME. We use the of PDL 1 expression status detected with 9A11 clone Abs and for IHC Sore for the densities of different subgroups of immune cells for statistical analysis. (PNG 187 kb) [file 12308_2023_530_Fig16_ESM.png]

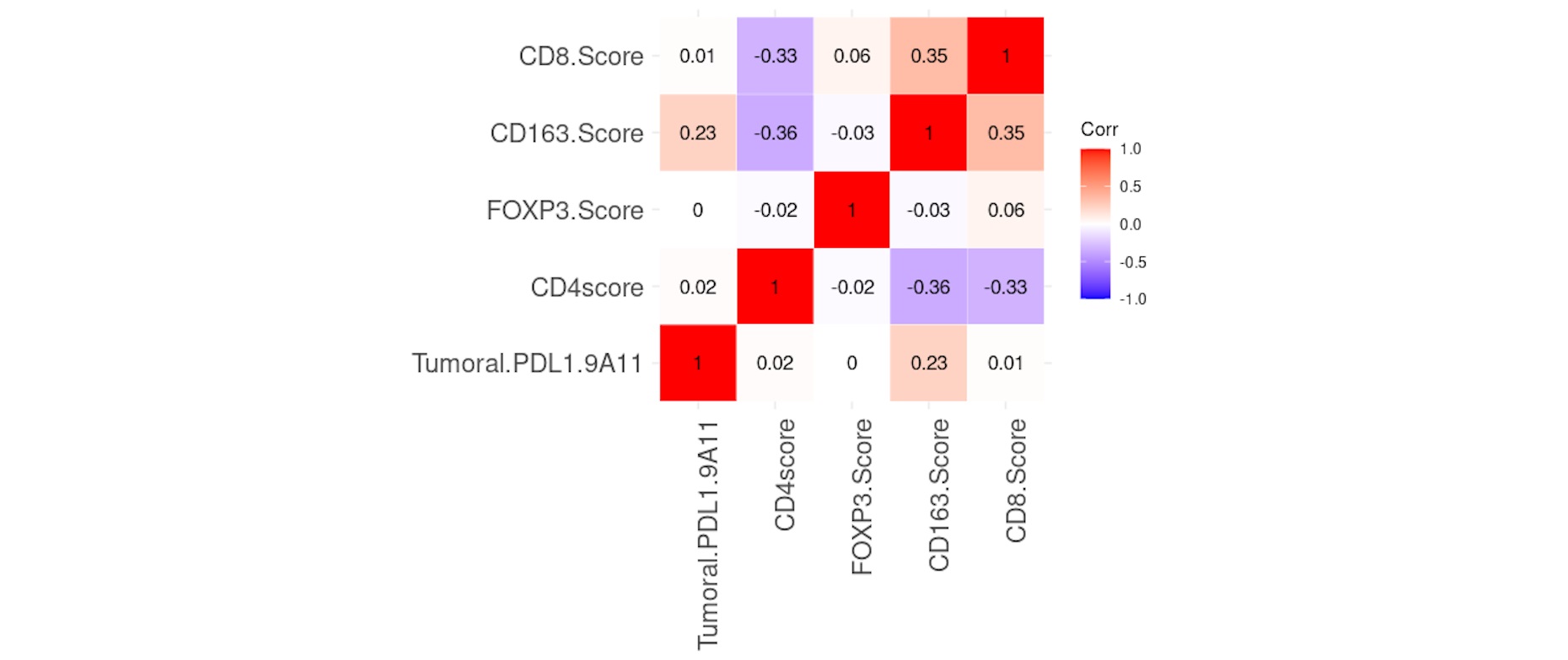

Supplement: Supplementary file 22 — High Resolution Image (TIF 95 kb) [file 12308_2023_530_MOESM11_ESM.tif]

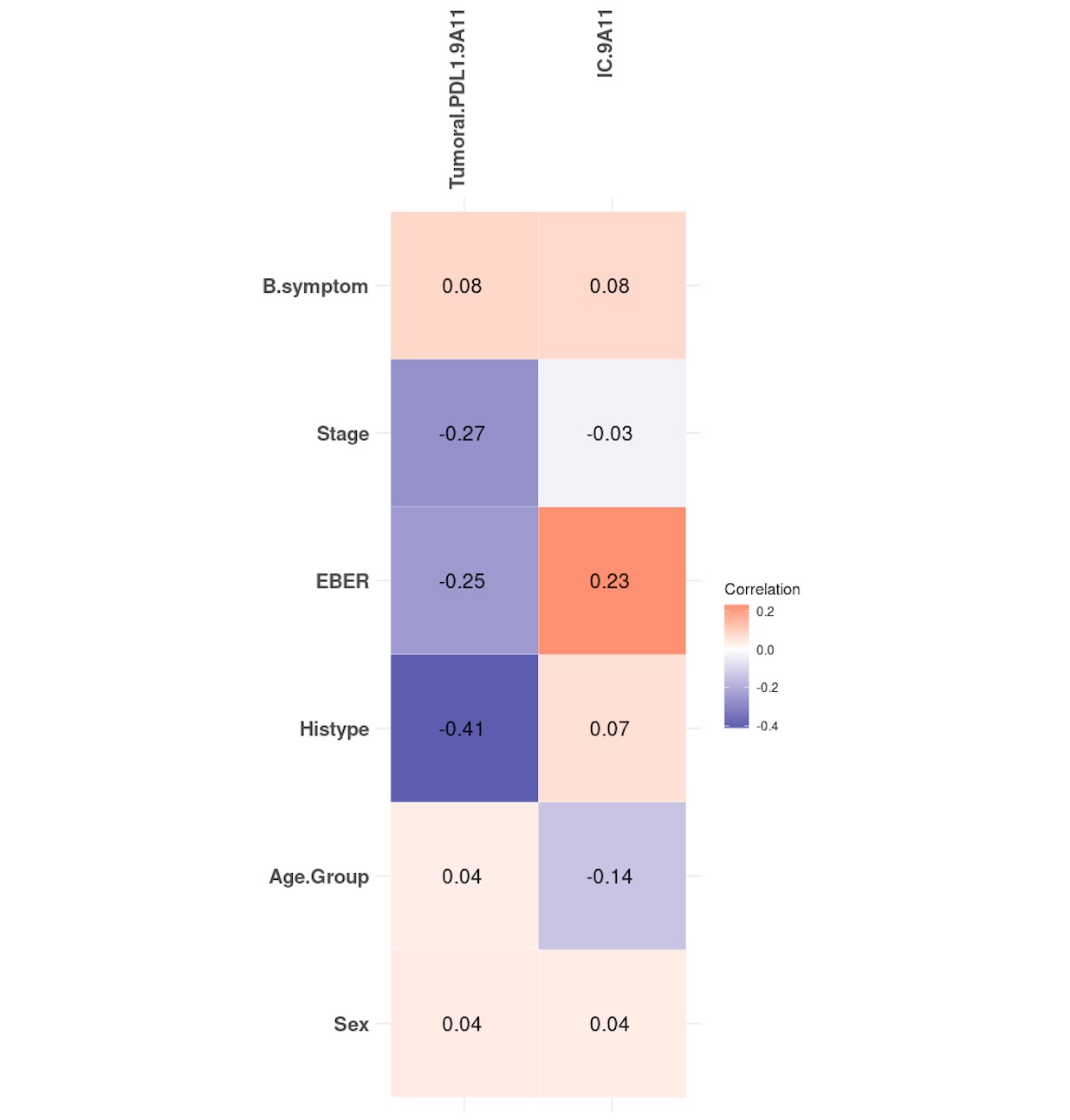

Supplement: Supplementary file 23 — Correlations between the PD-L1 expression clinicopathological parameters. Statistically analysis was done for correlation between PDL1 expression on CHL cells (HRS cells /background IC cells) and the clinicopathological parameters (sex, age, B symptoms, stage, pathology, EBER et al), detected by IHC with 9A11 clone Abs (JPG 70 kb) [file 12308_2023_530_MOESM12_ESM.jpg]

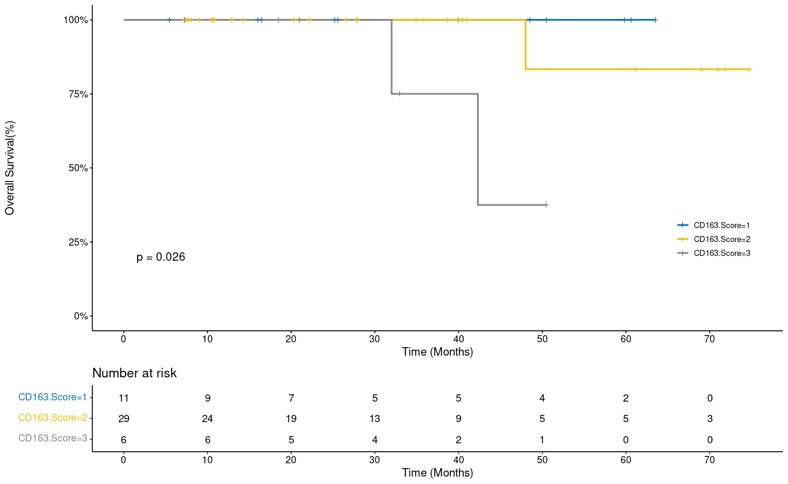

Supplement: Supplementary file 24 — Overall survival and event free survival analysis for the impact of CD163+ tumor associated macrophages and PD-L1 expression on IC. CHL cases were grouped according to CD163+ tumor associated macrophages density, as 1+:<5%, 2+:5-25%，and 3+: >25%) in Figure S4A and4B; and was groups according to PD-L1 expression level on immune cells (ICs) with 9A11 assay in Figure S4C and 4D, the cut off value as >25% named “high-IC”, or else “low-IC”), All were with univariable survival analysis, Kaplan-Meier method. (JPG 27 kb) [file 12308_2023_530_MOESM13_ESM.jpg]

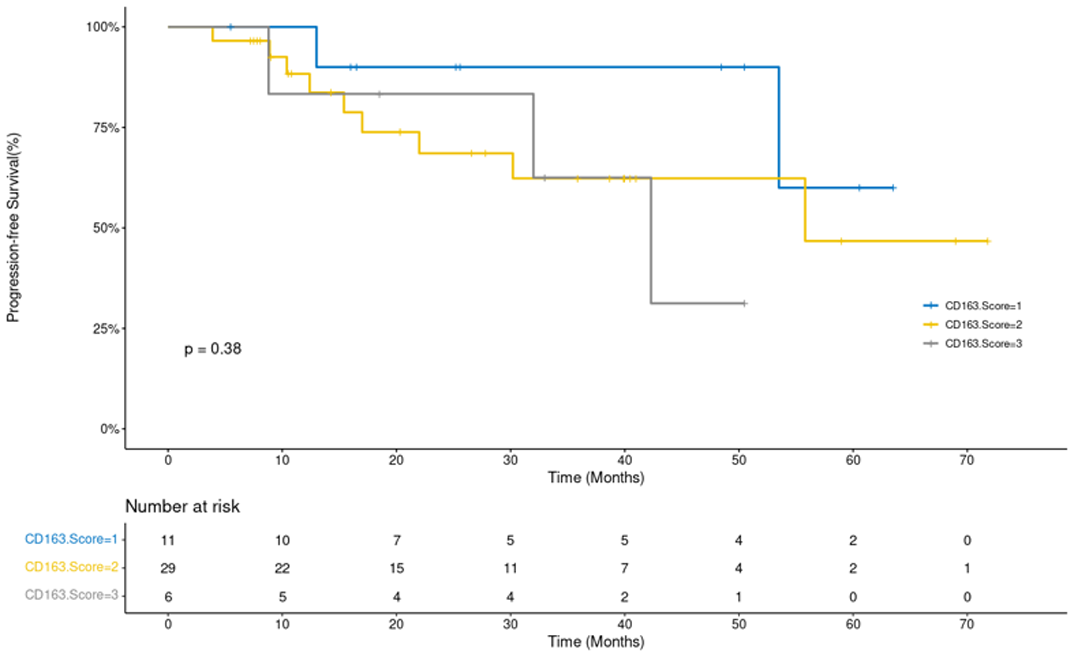

Supplement: Supplementary file 25 — (PNG 62 kb) [file 12308_2023_530_Fig17_ESM.png]

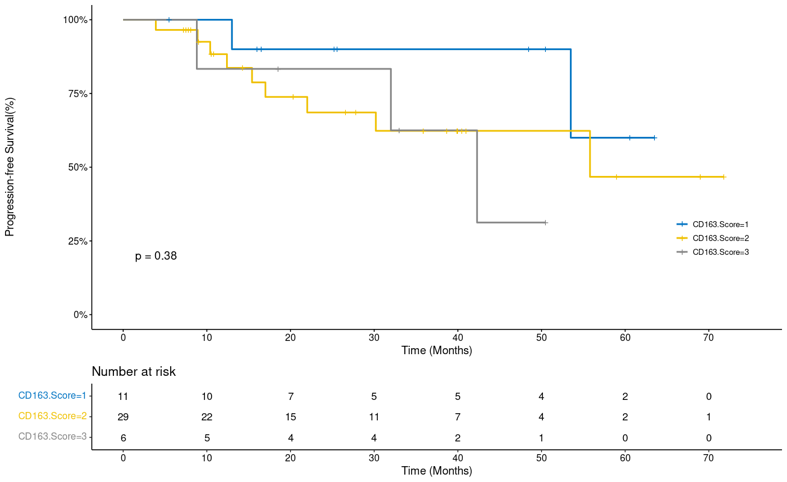

Supplement: Supplementary file 26 — High Resolution Image (TIF 50 kb) [file 12308_2023_530_MOESM14_ESM.tif]

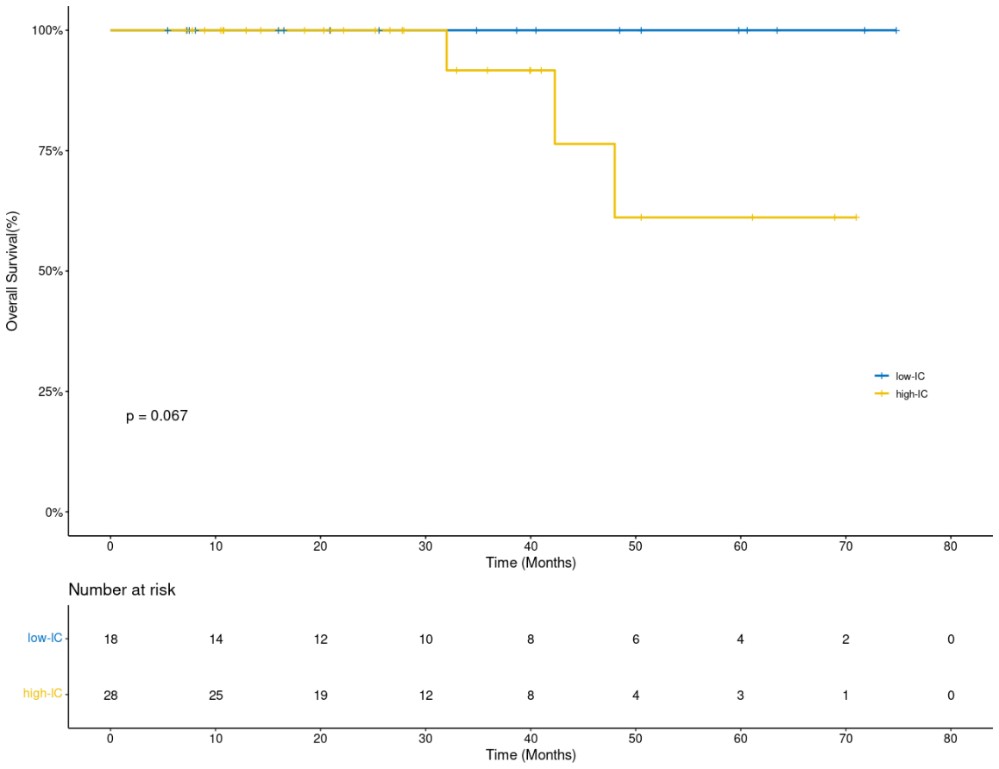

Supplement: Supplementary file 27 — (JPG 41 kb) [file 12308_2023_530_MOESM15_ESM.jpg]

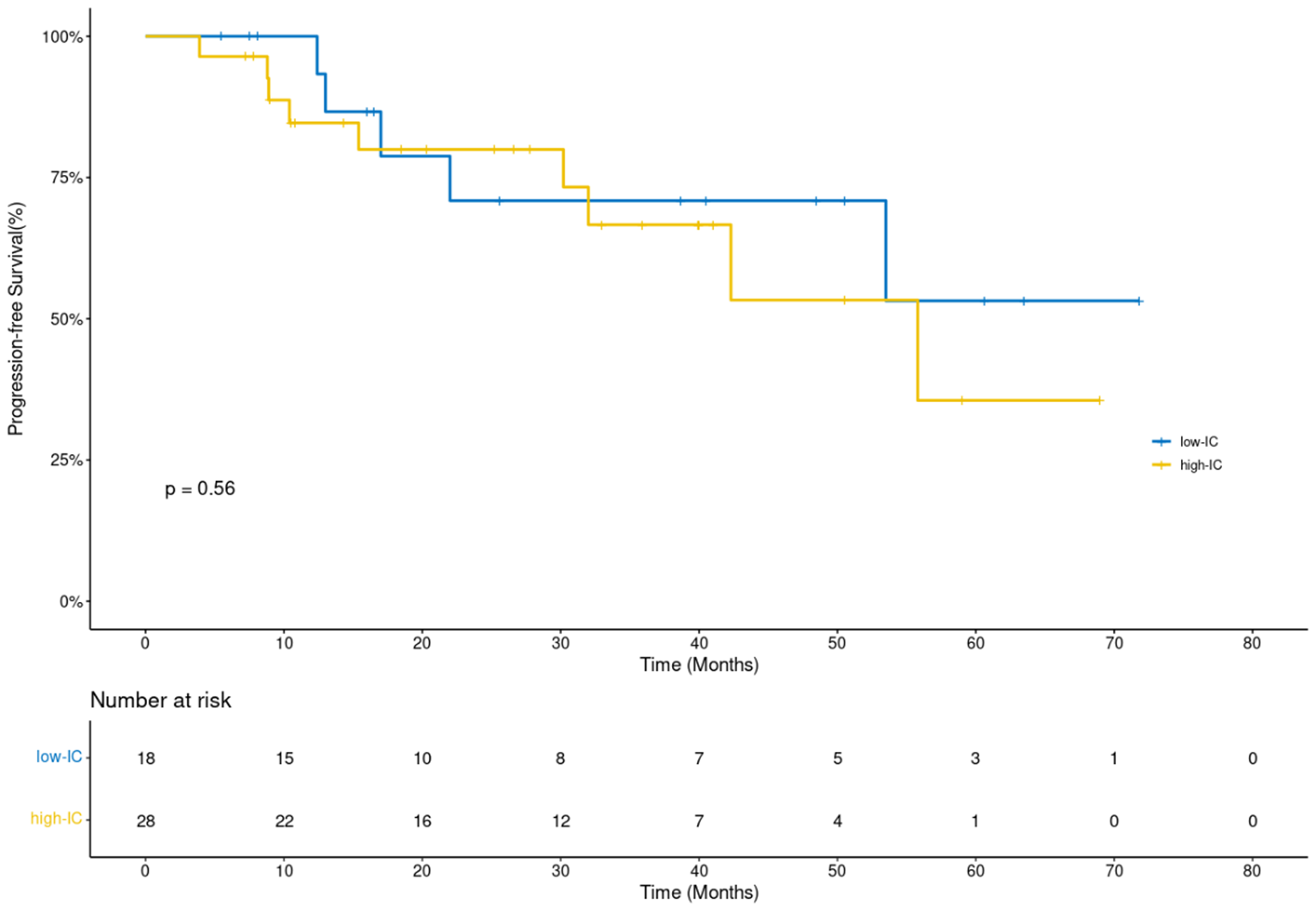

Supplement: Supplementary file 28 — (PNG 96 kb) [file 12308_2023_530_Fig18_ESM.png]

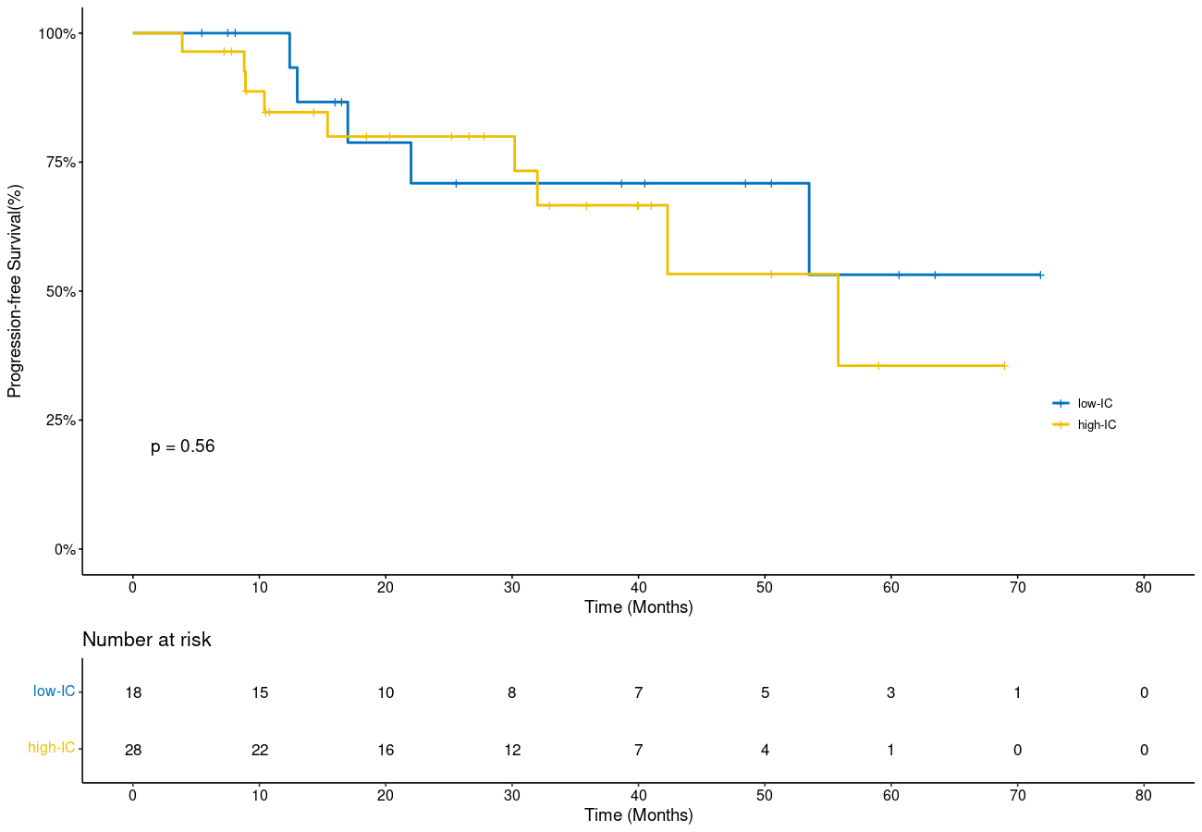

Supplement: Supplementary file 29 — High Resolution Image (TIF 102 kb) [file 12308_2023_530_MOESM16_ESM.tif]
