# Supplementary material for: PD-L1 immunohistochemistry assay optimization to provide more comprehensive pathological information in classic Hodgkin lymphoma
Source: J Hematop. 2023 Feb 1;16(1):7–16. doi: 10.1007/s12308-023-00530-1 (PMC10766715; doi:10.1007/s12308-023-00530-1)
Supplement: Supplementary file 30 — (DOCX 16 kb) [file 12308_2023_530_MOESM17_ESM.docx]

**Supplementary Table**

**Table S1** Survival data of all different subtypes of IC cells and CPS with different Abs for overall survival (OS) and PFS

|  | **For OS** | | |  | **For PFS** | | |
| --- | --- | --- | --- | --- | --- | --- | --- |
| **Characteristics** | Hazard.Ratio | CI95 | P.value |  | Hazard.Ratio | CI95 | P.value |
| **IC.9A11** | 1.03 | 0.99-1.07 | 0.095 |  | 1.00 | 0.99-1.02 | 0.765 |
| **IC.SP142** | 1.03 | 0.99-1.07 | 0.160 |  | 1.00 | 0.98-1.02 | 0.977 |
| **IC.22C3** | 1.03 | 0.99-1.07 | 0.201 |  | 1.00 | 0.98-1.02 | 0.814 |
| **PD1** | 1.91 | 0.17-21.24 | 0.599 |  | 0.81 | 0.23-2.91 | 0.747 |
| **CD4 density score** | 0 | 0-Inf | 0.999 |  | 1.53 | 0.54-4.31 | 0.425 |
| **FOXP3 density Score** | 4.96 | 0.26-94.76 | 0.288 |  | 0.79 | 0.28-2.21 | 0.656 |
| **CD163 density Score** | 9.37 | 1.02-86.37 | 0.048 |  | 1.78 | 0.77-4.1 | 0.176 |
| **CD8 density Score** | 1200551433 | 0-Inf | 0.999 |  | 1.11 | 0.44-2.81 | 0.829 |
