# Supplementary material for: PD-L1 immunohistochemistry assay optimization to provide more comprehensive pathological information in classic Hodgkin lymphoma
Source: J Hematop. 2023 Feb 1;16(1):7–16. doi: 10.1007/s12308-023-00530-1 (PMC10766715; doi:10.1007/s12308-023-00530-1)
Supplement: Supplementary file 31 — methology&suvival analysis (DOCX 28 kb) [file 12308_2023_530_MOESM18_ESM.docx]

Immunohistochemistry
The TMA sections was stained with the anti-PD-L1 antibody S 405.9A11 (dluted1:200 as recommended) on a Benchmark XT auto Stainer (Ventana Medical System, Tucson, AZ), using standard antigen retrieval (CC1 buffer, pH8.0, #950-124, Ventana). UltraView Universal DAB Detection kit (#760-500, Ventana) was used according to the manufacturer’s instruction. Counterstaining was performed as part of the automated staining protocol using hematoxylin (#760-2021, Ventana). After staining, slides were washed in soapy water and distilled water, dehydrated in graded alcohol and xylene, mounted and cover slipped.

For the SP142 assay, were stained with anti–PD-L1 (SP142) rabbit monoclonal primary antibody on the BenchMark ULTRA (Ventana Medical Systems Inc., Tucson, AZ) staining platform using the OptiView DAB IHC Detection Kit and OptiView Amplification Kit (Ventana Medical Systems Inc., Tucson, AZ). Briefly, antigen retrieval was undertaken for 48 minutes, the primary antibody was applied for 16 minutes at 36°C, amplification was done for 8 minutes amplifier/8 minutes multimer, and samples were counterstained for 4 minutes with hematoxylin II and post counterstained for 4 minutes.

For 22C3 staining, it was performed using the Dako Autostainer Link 48 platform and an automated staining protocol validated for the PD-L1 IHC 22C3 pharmDx assay. Deparaffinization, rehydration, and target retrieval was performed in the PT Link (Dako PT100) using a 3-in-1 procedure. After incubation with the antibody 22C3, specimens were incubated with anti-mouse linker antibody specific to the host species of the primary antibody, and then were incubated with a ready-to-use visualization reagent consisting of secondary antibody molecules and horseradish peroxidase molecules coupled to a dextran polymer backbone. The enzymatic conversion of the subsequently added 3,3′-diaminobenzidine tetrahydrochloride chromogen followed by 3,3′-diaminobenzidine tetrahydrochloride enhancer resulted in precipitation of a visible reaction product at the site of antigen. The specimens were then counterstained with hematoxylin and cover slipped.

***Survival Analysis for PD-L1 expression and subgroup of ICs***

The follow-up of living patients (with or without events) was censored at their last follow-up date. Overall Survival (OS) was defined as the interval between the date of diagnosis to death for any cause. Event-free survival (EFS) was defined as the interval between the date of diagnosis and the date of disease progression, relapse, or death from any cause. Median follow-up time was estimated on overall survival by reverse Kaplan-Meier method. Kaplan-Meier estimator was used to estimate survival probability. Survival difference between groups was tested by log-rank test for statistical significance. Confidence interval of survival rate was calculated by Greenwood's formula. Cox hazard regression was performed by analysis of univariable and multivariable risk factors. All statistical tests were two-sided with an alpha level of 0.05 as the significance cutoff. All analyses were performed in statistical software R 4.1.3 (NYC, co.).

The last follow-up date for CHL were in April 1st, 2022, and the Overall Survival (OS) time ranged from 7.0 to 94.5 months. The 5-year expected OS rate was 87% and 5-year expected Event Free Survival (EFS) rate was 59%. Univariable survival analysis were done for the impacts of CD4+, FOXP3+，CD8+，CD163+ TAMs densities with the clinical outcomes, only CD163 higher density(grouped by <5%,5%-25%,>25%) indicated worse outcome for OS (P=0.026, Figure 4A). Patients with higher level of PD-L1 expression of ICs (cut off value>25%) will also have obvious adverse effect on OS, although without statistically significance (*P*=0.067, Figure 4B). However, no significant difference of PFS were identified (Figure S4C and S4D). Survival analysis details can be seen in **Table S1.**

Comment Patients with higher density (>25%) of TMA was adverse predictor of clinical outcome by univariate survival analysis, which was in concordance with previous report [1]. In concordance of the closest relationship between CD163 positive TAM density, patients with higher level of PD-L1 on the ICs was associated with apparently worse OS. In fact, the CD163 positive TAM represent M2 macrophage[1], increased number of tumor-associated macrophages was strongly associated with shortened survival[2] and was also found in various tumors including diffuse large B-cell lymphoma[3-5]. ncreased expression of PD-L1 on ICs was most associated with elevated densities of CD163 positive tumor associated macrophages (TAMs). In concordance with TAMs, may have negative effect on survival of CHLs.

Reference

1. Klein JL, Nguyen TT, Bien-Willner GA, Chen L, Foyil KV, Bartlett NL, Duncavage EJ, Hassan A, Frater JL, Kreisel F: **CD163 immunohistochemistry is superior to CD68 in predicting outcome in classical Hodgkin lymphoma**. *American journal of clinical pathology* 2014, **141**(3):381-387.

2. Steidl C, Lee T, Shah SP, Farinha P, Han G, Nayar T, Delaney A, Jones SJ, Iqbal J, Weisenburger DD *et al*: **Tumor-associated macrophages and survival in classic Hodgkin's lymphoma**. *The New England journal of medicine* 2010, **362**(10):875-885.

3. Kiyasu J, Miyoshi H, Hirata A, Arakawa F, Ichikawa A, Niino D, Sugita Y, Yufu Y, Choi I, Abe Y *et al*: **Expression of programmed cell death ligand 1 is associated with poor overall survival in patients with diffuse large B-cell lymphoma**. *Blood* 2015, **126**(19):2193-2201.

4. Li Z, Dong P, Ren M, Song Y, Qian X, Yang Y, Li S, Zhang X, Liu F: **PD-L1 Expression Is Associated with Tumor FOXP3(+) Regulatory T-Cell Infiltration of Breast Cancer and Poor Prognosis of Patient**. *Journal of Cancer* 2016, **7**(7):784-793.

5. Hollander P, Kamper P, Smedby KE, Enblad G, Ludvigsen M, Mortensen J, Amini RM, Hamilton-Dutoit S, d'Amore F, Molin D *et al*: **High proportions of PD-1(+) and PD-L1(+) leukocytes in classical Hodgkin lymphoma microenvironment are associated with inferior outcome**. *Blood Adv* 2017, **1**(18):1427-1439.
